# Prognostic Gene Signature for Squamous Cell Carcinoma with a Higher Risk for Treatment Failure and Accelerated MEK-ERK Pathway Activity

**DOI:** 10.3390/cancers13205182

**Published:** 2021-10-15

**Authors:** Bohai Feng, Kai Wang, Esther Herpel, Michaela Plath, Wilko Weichert, Kolja Freier, Karim Zaoui, Jochen Hess

**Affiliations:** 1 Department of Otorhinolaryngology, Second Affiliated Hospital of Zhejiang University School of Medicine, Hangzhou 310000, China; bohai.feng@zju.edu.cn; 2 Department of Otorhinolaryngology, Head and Neck Surgery, Heidelberg University Hospital, 69120 Heidelberg, Germany; Michaela.Plath@med.uni-heidelberg.de (M.P.); karim.zaoui@med.uni-heidelberg.de (K.Z.); 3 Department of Respiratory Medicine, Fourth Affiliated Hospital of Zhejiang University School of Medicine, Yiwu 322000, China; kaiw@zju.edu.cn; 4 Institute of Pathology, University Hospital Heidelberg, and NCT Tissue Bank, National Center for Tumor Diseases (NCT), 69120 Heidelberg, Germany; Esther.Herpel@med.uni-heidelberg.de; 5 Institute of Pathology, Technical University Munich (TUM), and German Cancer Consortium (DKTK) Partner Site, 80333 Munich, Germany; wilko.weichert@tum.de; 6 Department of Oral and Maxillofacial Surgery, Saarland University Hospital, 66421 Homburg, Germany; kolja.freier@uks.eu; 7 Molecular Mechanisms of Head and Neck Tumors, German Cancer Research Center (DKFZ), 69120 Heidelberg, Germany

**Keywords:** HNSCC, squamous cell carcinoma, prognostic classifier, genetic and epigenetic alterations, multi-omics analysis, MEK inhibitors, MEK-ERK signaling

## Abstract

**Simple Summary:**

Squamous cell carcinoma (SCC) is the most prevalent type of human cancer worldwide and represents the majority of head and neck tumors. As SCC from aerodigestive or genitourinary tracts share not only common etiology and histological features but also molecular patterns, the major objectives of this study were the establishment of a pan-SCC-related prognostic gene signature by an integrative analysis of multi-omics data and the elucidation of underlying oncogenic pathway activities as potential vulnerabilities for a more efficient and less toxic therapy. Our approach delivers a reliable molecular classifier to identify HNSCC and other SCC patients at higher risk for treatment failure with tumors characterized by a more prominent MAPK activity, who might benefit from a targeted treatment with MEK inhibitors.

**Abstract:**

Squamous cell carcinoma (SCC) is the most prevalent histological type of human cancer, including head and neck squamous cell carcinoma (HNSCC). However, reliable prognostic gene signatures for SCC and underlying genetic and/or epigenetic principles are still unclear. We identified 37 prognostic candidate genes by best cutoff computation based on survival in a pan-SCC cohort (*n* = 1334) of The Cancer Genome Atlas (TCGA), whose expression stratified not only the pan-SCC cohort but also independent HNSCC validation cohorts into three distinct prognostic subgroups. The most relevant prognostic genes were prioritized by a Least Absolute Shrinkage and Selection Operator Cox regression model and were used to identify subgroups with high or low risks for unfavorable survival. An integrative analysis of multi-omics data identified FN1, SEMA3A, CDH2, FBN1, COL5A1, and ADAM12 as key nodes in a regulatory network related to the prognostic phenotype. An in-silico drug screen predicted two MEK inhibitors (Trametinib and Selumetinib) as effective compounds for high-risk SCC based on the Cancer Cell Line Encyclopedia, which is supported by a higher p-MEK1/2 immunohistochemical staining of high-risk HNSCC. In conclusion, our data identified a molecular classifier for high-risk HNSCC as well as other SCC patients, who might benefit from treatment with MEK inhibitors.

## 1. Introduction

Squamous cell carcinoma (SCC) is the most common type of human cancer, which arises from epithelial tissues of the upper aerodigestive or angiogenital tracts, skin, or lung [1,2,3]. SCCs are diagnosed as a substantial fraction of head and neck (90%) [4], cervix (62%) [5], oesophagus (38%) [6], non-melanoma skin (20–30%) [7], as well as lung cancers (30%) [8], and share common histological features and certain risk factors, e.g., smoking, alcohol abuse, and human papillomavirus (HPV) infection [9,10]. A recent pan-cancer study based on The Cancer Genome Atlas (TCGA) demonstrated similar molecular patterns among SCCs of different origins, which were distinct from other cancer entities [11]. This and other studies highlighted common features in the mutational landscape, such as copy number alterations (CNAs) at chromosome 3q and 5p among others [11,12], in oncogenic pathways, including Ras/MAPK and PI3K signaling [9] as well as in the immune microenvironment [13,14,15]. However, previous pan-SCC studies have not addressed common prognostic gene signatures and underlying genetic and/or epigenetic principles. These gene signatures have the innovative potential to improve risk assessment for treatment failure and to predict potential vulnerabilities for targeted therapy.

The availability of multi-omics data on all major cancers enables the development of novel molecular classification algorithms that can either complement or replace the established organ- and tissue-based tumor typing [11]. The rationale behind such molecular reclassifications is that genetic and epigenetic alterations offer a more precise view on underlying oncogenic principles and often provide an improved predictive value for therapeutic response [11]. As a consequence, basket trials have been launched as a new clinical study design in which targeted therapy is evaluated for multiple cancers with common molecular patterns, but independent of their site of origin [16]. With the rising interest and effort toward precision oncology, it is vital to elucidate the prognostic biomarkers shared among tumors of distinct origins, enabling a better risk prediction for cancer patients, who will benefit from targeted therapies [17,18].

Hence, the main objectives of our study were the establishment of common prognostic gene signatures to enable the stratification of SCC patients at higher risk for treatment failure and to predict promising drug targets for a more effective and less toxic therapy. We identified three robust prognostic subtypes in a pan-SCC training cohort based on consensus clustering of the expression profile for 37 survival-related genes, which were confirmed in independent HNSCC validation cohorts. Subsequently, 18 candidate genes were prioritized by a Least Absolute Shrinkage and Selection Operator (LASSO) Cox regression algorithm to establish a prognostic risk model for SCC patients. Finally, we performed an integrative analysis of multi-omics data to unravel key nodes of a functional network, and proposed potential drug targets for high-risk SCC based on the *in-silico* drug screening analysis of SCC cell lines.

## 2. Results

### 2.1. Molecular Prognostic Subgroups in a Pan-SCC Cohort

Four individual gene expression datasets with 1334 SCC cases, including TCGA-CESC (Cervical squamous cell carcinoma and endocervical adenocarcinoma; *n* = 252), TCGA-ESCA (Esophageal carcinoma; *n* = 81), TCGA-HNSC (Head and Neck squamous cell carcinoma; *n* = 500), and TCGA-LUSC (Lung squamous cell carcinoma; *n* = 501) were used to identify prognostic candidate genes by best cutoff computation considering either overall survival (OS) or progression-free intervals (PFI) as the clinical endpoint. The transcription of 258 genes were significantly associated with OS in at least three out of four SCC datasets, including 145 candidate genes related to a favorable and 133 candidate genes related to an unfavorable OS. In addition, the transcription of 276 genes were significantly associated with PFI in at least three out of four datasets, including 153 candidate genes related to a favorable and 123 candidate genes related to an unfavorable PFI (Figure S1; Table S1). Gene Ontology (GO) enrichment analyses demonstrated an enrichment for genes implicated in DNA replication for the gene set related to a favorable OS, response to wounding for the gene set related to an unfavorable OS, and T cells differentiation and activation for the gene set related to favorable PFI (Figure S2). Finally, 37 survival-related candidate genes were identified, which were significantly correlated with an unfavorable (*n* = 20) or favorable (*n* = 17) prognosis concerning both OS and PFI, respectively (Table S2). Comparing matched tumor samples and normal tissue, we found twelve of these candidate genes to be differential expressed (−1 > log_2_FC > 1, FDR < 0.05) in the pan-SCC cohort, 14 in the TCGA-HNSC, and 16 in the TCGA-LUSC cohorts (Figure S3). However, it is worth noting that up- or down-regulation of survival-related candidate genes among matched normal versus tumor tissue was not strictly related with their association concerning a favorable or unfavorable prognosis (Figure S3). As an example, five candidates were up-regulated in SCCs as compared to normal tissue of which three (*PLOD1*, *PLOD3*, and *SLC16A3*) also shared a higher expression in tumors with an unfavorable prognosis, while a higher expression of two candidate genes (*FOXRED2* and *FOXE1*) were associated with a favorable outcome (Figure S3A).

**Figure 1:**
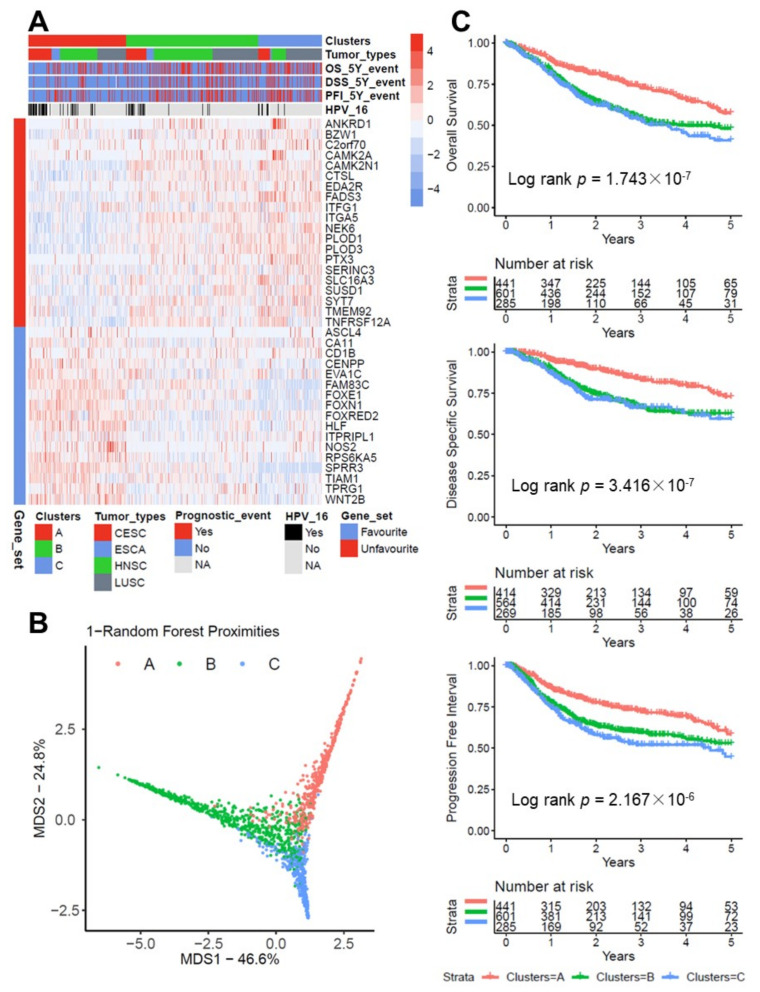
Establishment of a prognostic pan-SCC classifier based on survival-related candidate genes. (**A**) Heatmap illustrates classification of pan-SCC cases by k-means clustering based on a distance matrix calculation from the resampled expression data of indicated candidate genes. Rows and columns indicate candidate genes and cases, respectively. (**B**) Random forest MDS plot presents distinct features of clusters based on 37 survival-related candidate genes for the pan-SCC cohort. (**C**) Kaplan–Meier plots for five-years OS (top), DSS (middle), and PFI (bottom) of the three clusters for the pan-SCC cohort. *p*-values were computed by the log-rank test and numbers represent cases at risk at the indicated time points.

Consensus clustering of 1334 SCC cases based on the expression profile of 37 survival-related candidate genes revealed three robust clusters (Figure 1A and Figure S4), which were further supported by a random forest regression model for the pan-SCC cohort (Figure 1B). The SCC cases in cluster A (*n* = 444) were characterized by a higher expression of candidate genes related to a favorable survival, while cluster B (*n* = 602) and C (*n* = 288) shared a higher expression of candidate genes related to an unfavorable survival (Figure 1A). Moreover, cluster A was significantly enriched for HPV16-positive SCC as compared to cluster B and C (Chi-square test *p* value < 0.001).

In terms of the clinical outcome, patients of cluster A exhibited an improved survival as compared to clusters B and C, which reached statistical significance for five-years OS, disease specific survival (DSS), and PFI in the pan-SCC cohort (Figure 1C). Univariate Cox regression models confirmed a significantly favorable clinical outcome (OS, DSS, and PFI) for cluster A in all cohorts except TCGA-ESCA, which was independent of the HPV16 status (Figure S5).

The robustness of the 37 gene signature to stratify three prognostic clusters was confirmed for two independent HNSCC cohorts (GSE65858 [19] and GSE117973 [20]) and one HPV-negative oral SCC (GSE41613 [21]) cohort (Figure S6). Again, HNSCCs in cluster A had a significantly favorable OS as compared to cluster B and C for the combined validation cohort, which was also evident in the subgroup of HPV-negative HNSCC (Figure S7).

### 2.2. Prioritization of Prognostic Candidate Genes by a LASSO Cox Regression Model

**Figure 2:**
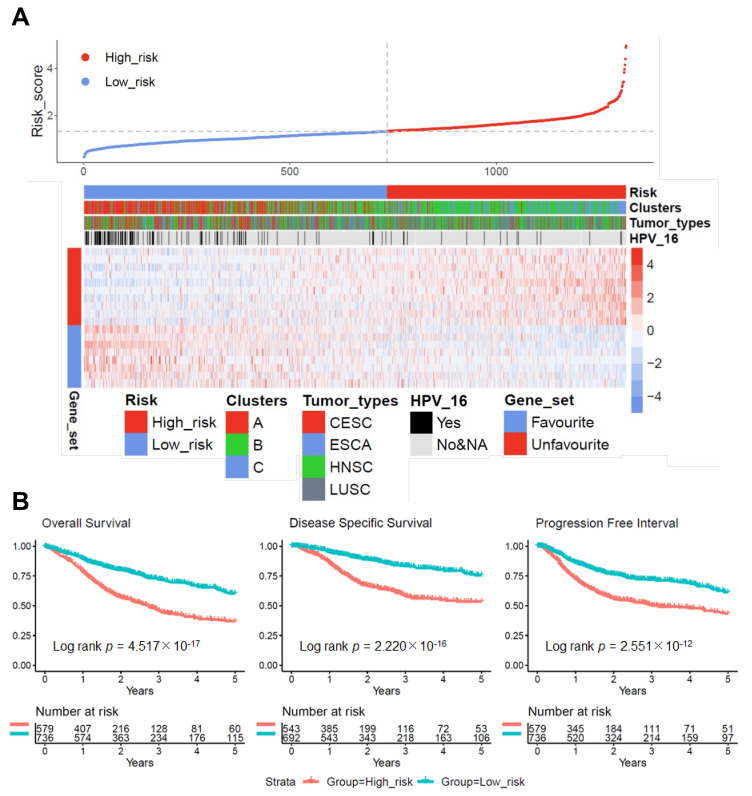
LASSO Cox regression model for the pan-SCC cohort. (**A**) Dot plot displays risk scores for low-risk (blue dots) or high-risk (red dots) subgroups, and the heatmap summarizes expression of 18 prioritized candidate genes within the pan-SCC cohort. (**B**) Kaplan–Meier plots for five-year OS (left), DSS (middle), and PFI (right) of the low-risk and high-risk subgroups for the pan-SCC cohort. *p*-values were computed by the log-rank test and numbers represent cases at risk for the indicated time points.

Most relevant prognostic candidate genes were prioritized by a LASSO Cox regression model concerning OS in 1315 cases of the pan-SCC cohort (Figure S8). The analysis revealed 18 prognostic candidate genes, of which seven were related to a favorable OS (RPS6KA5, EVA1C, FOXRED2, ITPRIPL1, TIAM1, FAM83C, and NOS2), and eleven were related to an unfavorable OS (BZW1, CTSL, TPRG1, ITGA5, SLC16A3, PTX3, CAMK2A, SERINC3, SUSD1, EDA2R, and TMEM92). The pan-SCC cohort was divided into high-risk (*n* = 579) and low-risk (*n* = 736) groups based on the risk score best cutoff by a log rank analysis. As expected, cluster A cases were strongly enriched in the low-risk group, while cluster B and C cases were enriched in the high-risk group (Figure 2A). Moreover, HPV-positive tumors and cases of the TCGA-CESC cohort were more abundant in the low-risk group (Figure 2A). Concerning the clinical outcome, the high-risk group of the pan-SCC cohort was associated with an unfavorable OS, DSS, and PFI as determined by a Kaplan–Meier analysis (Figure 2B), and univariate Cox regression models confirmed an unfavorable OS for most tested subgroups with a high-risk score (Figure S9). Moreover, univariate Cox regression models confirmed an unfavorable OS, DSS, and PFI for HPV16-positive and HPV-16 negative SCC with a high-risk score for TCGA-CESC or TCGA-HNSC, but not for HPV18-positive SCC of TCGA-CESC (Figure S10).

Concerning the impact of the tumor immune microenvironment (TIME), we did not observe a significant correlation among the prognostic clusters or risk groups with a previously reported cytotoxic immune phenotype [15] (data not shown). Furthermore, the analysis of ESTIMATE signatures (stromal, immune, and ESTIMATE scores) revealed no significant difference among high-risk and low-risk groups in TCGA-CESC or TCGA-ESCA cohorts, while significant differences were detected for TCGA-LUSC and in part for the TCGA-HNSC cohorts (Figure S11).

### 2.3. Differences in the Mutational Landscape Related to Prognostic Risk Groups

**Figure 3:**
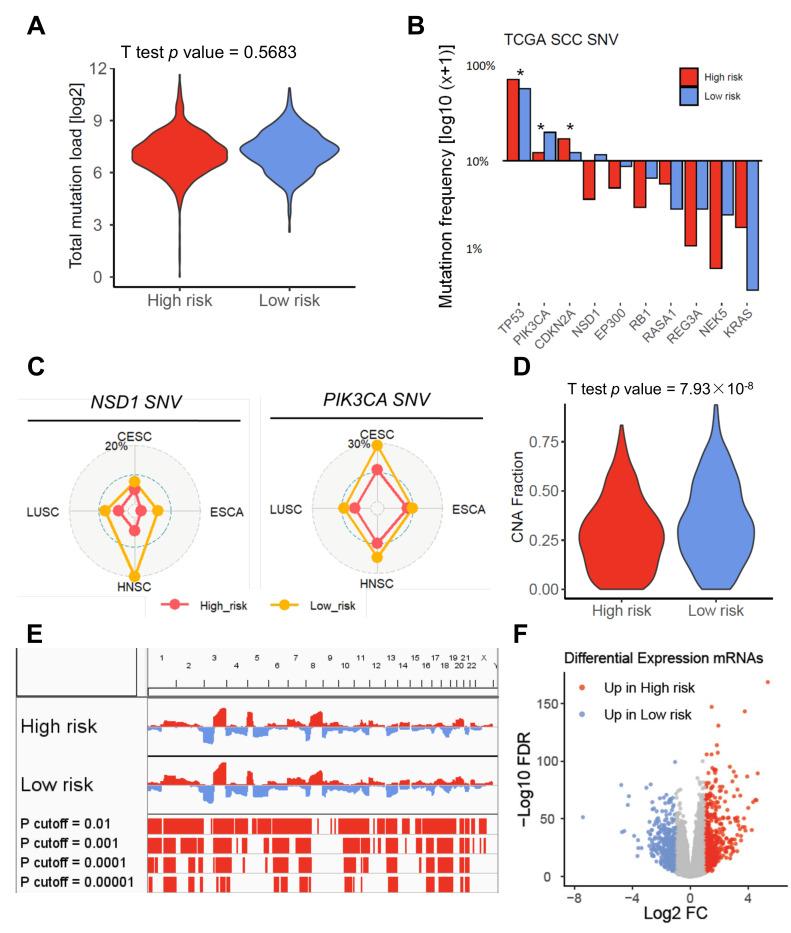
Differences in the mutational landscape among high-risk and low-risk subgroups of the pan-SCC cohort. (**A**) Violin plot demonstrates no significant difference for total mutation load among high-risk and low-risk subgroups of the pan-SCC cohort. (**B**) Bar plot summarizes the relative abundance of somatic mutations for significant MutSig genes (chi-square test *p* < 0.05) among high-risk and low-risk subgroups of the pan-SCC cohort. * chi-square test *p* < 0.001. (**C**) Graphs show the relative frequency of either NSD1 (left) or PIK3CA (right) somatic mutations for the indicated TCGA cohorts. (**D**) Violin plot demonstrates a significant higher global CNA fraction in the low-risk as compared to high-risk subgroup for the pan-SCC cohort. (**E**) CNA plot shows the relative frequency of copy number gains (red) or deletions (blue) among high-risk and low-risk subgroups of the pan-SCC cohort and displays highly significant differences by Fisher’s exact test. (**F**) Volcano plot presents significant differentially expressed genes (DEGs) (−1 > log_2_FC > 1, FDR < 0.05, *n* = 1081) among high-risk and low-risk subgroups of the pan-SCC cohort computed by edgeR.

To address the question of whether distinct prognostic phenotypes are the consequence of a complex regulation of molecular networks, including genetic and/or epigenetic events, we conducted an integrative analysis of multi-omics data. First, we analyzed the quantity and quality of somatic mutations among low-risk and high-risk groups of the pan-SCC cohort. While no significant difference for the total mutation load was evident in the pan-SCC cohort (Figure 3A) or for individual SCC cohorts (Figure S12A), the relative frequency of NSD1 and PIK3CA mutations was significantly higher in SCCs of the low-risk as compared to the high-risk group (Figure 3B). In contrast, the relative frequency of TP53 mutations was significantly higher in tumors of the high-risk as compared to the low-risk group. While differences in the relative frequency of NSD1 and PIK3CA mutations were consistent for all analyzed SCC cohorts (Figure 3C), the enrichment of TP53 mutations in the high-risk group was particularly detected for TCGA-HNSC (Figure S12B).

In terms of CNAs, we identified a significantly higher fraction of global CNAs in the low-risk as compared to high-risk group of the pan-SCC cohort (Figure 3D), which was significant for HPV16-negative SCC and TCGA-LUSC, but not any other subgroup (Figure S13). An analysis of the quality of CNAs revealed several hot spot regions with copy number gains (chromosomes 1q, 3q, 7q, 18p, and 18q) or deletions (chromosomes 6q, 10q, 13q, and 16q) as characteristic features of the low-risk group in the pan-SCC cohort (Figure 3E). Several of these hot spot regions were also observed in distinct SCC subtypes, in particular TCGA-LUSC (Figure S14). To evaluate whether these CNAs explain at least in part changes in global gene expression, differentially expressed genes (DEGs) between low-risk and high-risk groups of the pan-SCC cohort were analyzed. In total, 1081 DEGs (−1 > log_2_FC > 1 and FDR < 0.05) were identified, including 520 genes with higher transcript levels in the low-risk group and 561 genes with a higher transcript level in the high-risk group (Figure 3F; Table S3). Eight up-regulated DEGs in the high-risk group were related to the unfavorable gene set considering OS and PFI (ANKRD1, CAMK2A, CAMK2N1, FADS3, ITGA5, PTX3, SYT7, and TNFRSF12A), while six up-regulated DEGs in the low-risk group were related to the favorable gene set considering OS and PFI (FOXE1, FOXN1, FOXRED2, HLF, NOS2, and SPRR3). An enrichment analysis considering MSigDB hallmark, C2, C5, and C6 datasets revealed extracellular matrix organization and interaction, epithelial mesenchymal transition, invasion, coagulation, BMI, KRAS, and EGFR signaling as the top features for SCC of the high-risk group, and distinct metabolic processes, E2F activity, G2M checkpoint, HPV positivity, and epithelial differentiation as the top features for SCC of the low-risk group (Tables S4–S7). In total, 49 up-regulated DEGs in SCCs of the high-risk group were encoded by genomic regions with a significantly higher frequency for deletions in SCCs of the low-risk group, while 68 up-regulated DEGs in SCCs of the low-risk group were encoded by genomic regions with a significantly higher frequency for copy number gains in this group (Table S8).

In summary, these data indicate an impact of specific somatic mutations and CNAs on the clinical outcome of SCCs. It is worth noting that previous studies already demonstrated a favorable survival of HNSC with NSD1 mutations accompanied by global DNA hypomethylation [22,23], suggesting a pivotal role of epigenetic events for the prognosis of SCCs.

### 2.4. Differences in Epigenetic Events Related to Prognostic Risk Groups

**Figure 4:**
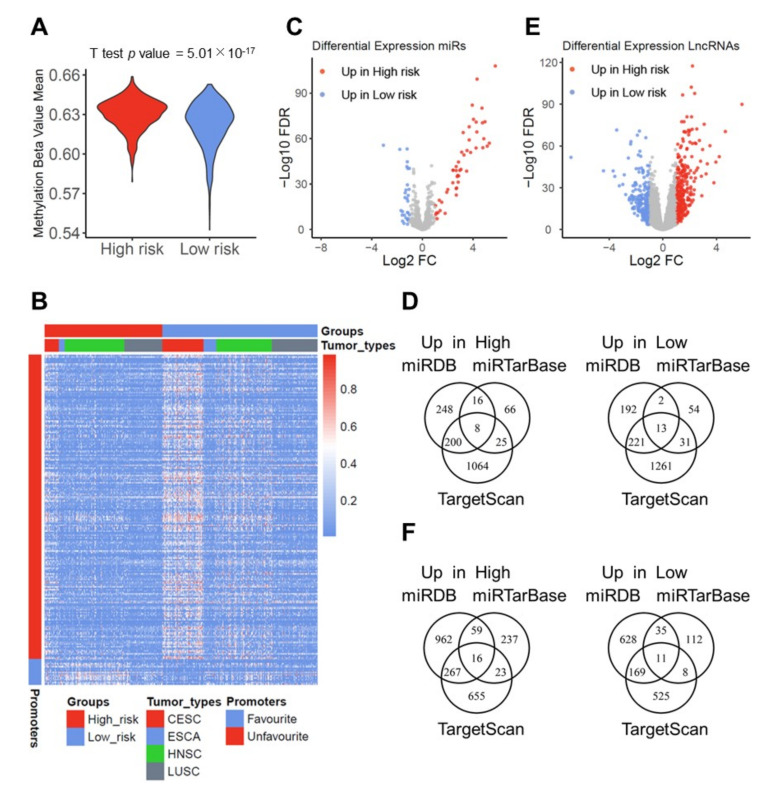
Differences in the epigenetic landscape among high-risk and low-risk subgroups of the pan-SCC cohort. (**A**) Violin plot demonstrates significant higher genome-wide beta mean values for promoter DNA methylation of the low-risk as compared to the high-risk groups in the pan-SCC cohort. (**B**) Heatmap shows beta values for differentially methylated probes (*n* = 231) annotated for promoters of DEGs among high-risk and low-risk subgroups of the pan-SCC cohort. (**C**) Volcano plot shows significant differentially expressed miRs (DEFMs, *n* = 78) among high-risk and low-risk groups of the pan-SCC cohort (−1 > log_2_FC > 1, FDR < 0.05). (**D**) Venn diagrams summarize the amount of predicted DEFM-DEG links according to indicated databases and miRs which are up-regulated in either high-risk (left) or low-risk (right) groups of the pan-SCC cohort. (**E**) Volcano plot shows significant differentially expressed lncRNAs (*n* = 513) among high-risk and low-risk groups of the pan-SCC cohort (−1 > log_2_FC > 1, FDR < 0.05). (**F**) Venn diagrams summarize the amount of predicted miR-DEG links according to indicated databases and based on miRs, which are linked to differentially expressed lncRNAs, which are up-regulated in either the high-risk group (left) or the low-risk group of the pan-SCC cohort.

To explore the impact of DNA methylation on the establishment and maintenance of prognostic risk groups, we analyzed global DNA methylation data of the pan-SCC cohort. The global methylation value was significantly lower in SCCs of the low-risk as compared to the high-risk group (Figure 4A), which was consistently found for HPV16-negative and HPV16-positive subgroups or distinct SCC subtypes, except for TCGA-ESCA (Figure S15A–B). Furthermore, 4503 differentially methylated probes (−0.5 > log_2_FC > 0.5, FDR < 0.05) were extracted comparing low-risk and high-risk groups of the pan-SCC cohort (Figure 4B). A total of 213 probes had significantly higher beta values for the low-risk group and were located in proximal promoters of 66 DEGs with higher expression in SCCs of the high-risk group. Finally, 18 probes had significantly higher beta values for the high-risk group and were located in the proximal promoter of nine DEGs with a higher expression in SCCs of the low-risk group (Table S9). Significant differences in beta mean values of probes in the proximal promoter of DEGs related to favorable or unfavorable survival were also found for distinct tumor entities (Figure S16A,B).

We also identified 78 differentially expressed functional miRNAs (DEFM) (−1 > log_2_FC > 1, FDR < 0.05) among low-risk and high-risk groups for the pan-SCC cohort (Figure 4C; Table S10). For miRNAs with higher expression in the high-risk group, 267 DEFM-DEG links were identified by at least two databases, including 170 up-regulated DEGs in SCCs of the low-risk group. For miRNAs with higher expression in the low-risk group, 249 DEFM-DEG links were identified, including up-regulated 160 DEGs in SCCs of the high-risk group (Figure 4D).

Next, we identified 513 differentially expressed lncRNAs (−1 > log_2_FC > 1, FDR < 0.05) among low-risk and high-risk groups of the pan-SCC cohort (Figure 4E, Table S11). The target miRNA prediction revealed 2105 lncRNA-miR links, including 41 lncRNAs and 275 miRNAs, of which 363 target 175 up-regulated DEGs in SCCs of the high-risk group and 223 target 127 up-regulated DEGs in SCCs of the low-risk group (Figure 4F). Based on the differentially expressed miRs and lncRNAs as well as predicted links with targeted DEGs, a complex network was plotted that summarizes the underlying molecular traits for distinct prognostic risk profiles of the pan-SCC cohort (Figure S17).

### 2.5. DEGs Affected by Several Modes of Genetic and Epigenetic Regulation

**Figure 5:**
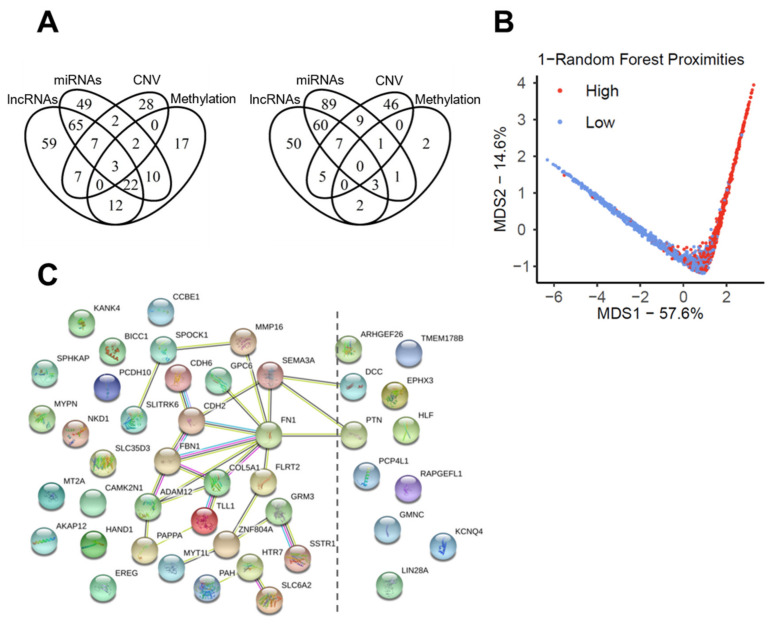
Candidate genes affected by different modes of genetic and epigenetic regulation. (**A**) Venn diagrams show the amount of DEGs with higher expression in either the high-risk (left) or the low-risk (right) group of the pan-SCC cohort, which are affected by different modes of indicated genetic (CNV) or epigenetic (Methylation, miRs, lncRNAs) events. (**B**) Random forest MDS plot presents distinct features of risk groups based on DEGs (*n* = 45), which are affected by three out of four genetic and epigenetic events. (**C**) Schematic presentation of a protein–protein interaction network (minimum required interaction score: medium confidence (0.400)) for selected DEGs (*n* = 45), which are affected by at least three out of four genetic or epigenetic events according to the STRING database.

We assumed that DEGs regulated by different modes of genetic and epigenetic events might represent important drivers of our prognostic risk model. In total, 45 DEGs were identified, which were affected in at least three out of four genetic or epigenetic models (Table S12). Of these DEGs, two (CAMK2N1 and HLF) were previously identified as candidate genes of the prognostic 37 gene signature, 34 shared a higher expression in SCCs of the high-risk group and eleven were up-regulated in SCCs of the low-risk group (Figure 5A). A random forest regression model confirmed the stratification of tumors in the pan-SCC cohort into low-risk and high-risk groups based on the transcript levels of these 45 DEGs (Figure 5B). In addition, the analysis of protein–protein interaction according to the STRING database highlighted FN1, SEMA3A, CDH2, FBN1, COL5A1, and ADAM12 as key nodes of a well-defined network (Figure 5C). Interestingly, the prominent expression of these key nodes was detected in either fibroblasts or cancer cells with an mesenchymal-like phenotype in HNSCC (Figure S18), according to single-cell RNA sequencing data (GSE103322) [24].

### 2.6. In-Silico Drug Screen for SCC Cell Lines Resembling a High or Low-Risk Profile

**Figure 6:**
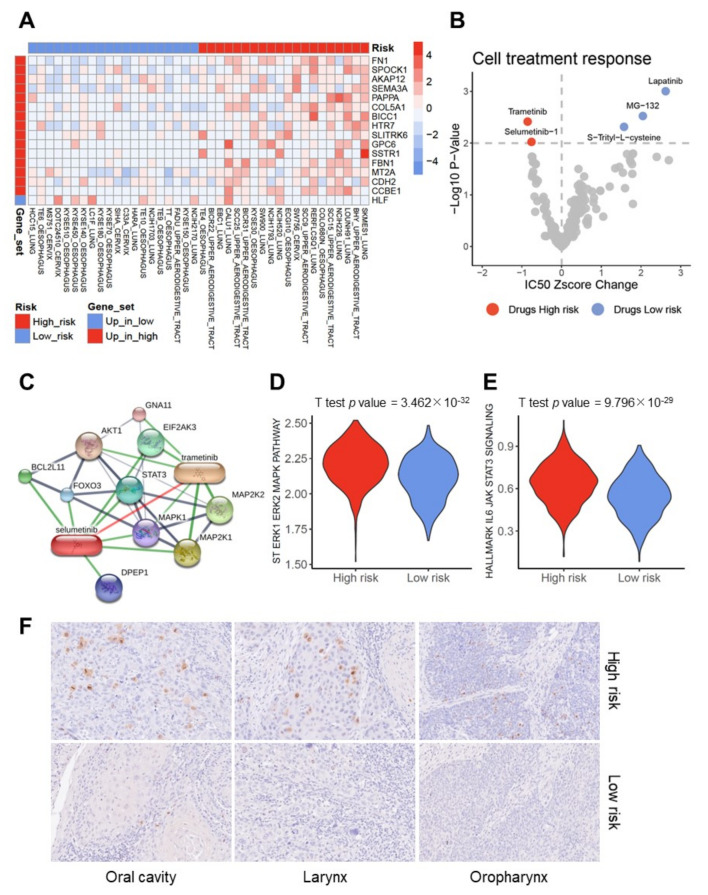
MEK inhibitors as potential drugs for high-risk SCC. (**A**) Heatmap demonstrates expression pattern of 16 survival-related candidate genes in indicated cancer cell lines with a low-risk or high-risk profile. (**B**) Volcano plot summarizes the relative change in the IC50 z-score and *p*-value among selected SCC cell lines with a low-risk or high-risk profile, which were treated with indicated compounds based on the Genomics of Drug Sensitivity in Cancer (GDSC) database. Red dots represent drugs with a significantly higher sensitivity for high-risk SCC cell lines and blue dots represent drugs with a significantly higher sensitivity for low-risk SCC cell lines. (**C**) Schematic presentation of a protein–protein and protein–chemical association network (minimum required interaction score: medium confidence (0.400)) for Trametinib and Selumetinib based on the STITCH database. (**D**,**E**) Violin plots demonstrate significantly higher ssGSEA scores for either ERK-MAPK or JAK-STAT3 signaling pathways in high-risk as compared to low-risk groups of the pan-SCC cohort. (**F**) Representative pictures of an IHC staining demonstrates prominent MEK1/2 phosphorylation (brown signal) in cancer cells of high-risk (upper row) but not in low-risk (lower row) HNSCC of the indicated subsites. Histological staining with hematoxylin to visualize the tissue architecture.

Finally, we addressed the question of whether SCC cells with a high-risk profile share traits of resistance or sensitivity to well-established compounds with the perspective to elucidate vulnerabilities for a more effective treatment of high-risk SCC patients. Risk scores were computed for 66 tumor cell lines from cervix, oesophagus, upper aerodigestive tract, and lung SCCs based on the previously established 18 gene signature. SCC cell lines with the highest or lowest risk scores (*n* = 20, respectively) were selected for further analysis (Figure S19A). It is worth noting that the selected SCC cell lines exhibited a significant difference in transcript levels for 16 out of 45 candidate genes, which were affected by genetic or epigenetic alterations in the pan-SCC cohort (Figure 6A and Figure S19B). In line with the pan-SCC cohort, SCC cell lines with a low-risk profile had a significantly higher CNA fraction as compared to cell lines with a high-risk profile (Figure S20A), accompanied by a significantly higher frequency of chromosome 3q gains (Figure S20B). Again, no statistically significant difference was evident concerning total mutational load (Figure S20C), but a trend towards a higher frequency of RB1 somatic mutations was found for SCC cell lines with a low-risk profile, while more KRAS somatic mutations were present in cell lines with a high-risk profile (Figure S20D). We detected a difference in neither the frequency of NSD1 somatic mutations nor in the global beta mean value for DNA methylation among SCC cell lines with a low-risk or high-risk profile (Figure S20D,E). However, the beta mean value of probes in the proximal promoter of up-regulated DEGs in the high-risk group of the pan-SCC cohort was significantly higher in SCC cell lines with the low-risk profile (Figure S20F).

Drug sensitivity data of the CCLE database were analyzed and revealed a significantly higher sensitivity for SCC cell lines with a high-risk profile under Trametinib and Selumetinib-1 treatment, while SCC cell lines with a low-risk profile were more sensitive to Lapatinib, MG-132, or *S*-Trityl-l-cysteine (Figure 6B). Trametinib and Selumetinib-1 are well established MEK inhibitors [25,26], and according to the protein–chemical association network based on the STITCH database, are related to MEK-ERK and STAT3 signaling (Figure 6C). Indeed, significantly higher ssGSEA scores for either MAPK-ERK or STAT3 signaling were detected for SCC cell lines with a high-risk as compared to a low-risk profile (Figures S21A and S22A) of the pan-SCC cohort (Figure 6D,E). The MAPK-ERK pathway was consistently enriched in the high-risk group independent of the HPV16 status or the SCC subtype, except for TCGA-ESCA (Figure S21B,C). Significantly higher ssGSEA scores for the JAK-STAT3 pathway were evident for HPV16-negative tumors of the pan-SCC cohort, but not for HPV16-positive SCC (Figure S22B). Concerning individual SCC subtypes, significantly higher ssGSEA scores for the JAK-STAT3 pathway were confirmed for TCGA-HNSC and TCGA-LUSC (Figure S22C). These data suggest a pivotal role of MEK1/2 activation for the prognosis of SCC, which is further supported by significantly higher MEK1 (pS217/221) phosphorylation levels, but not total protein amounts in the high-risk group of the pan-SCC cohort according to The Cancer Proteome Atlas (TCPA) (Figure S23A). In addition, significant higher levels of the up-stream signaling proteins CRAF and phospho-EGFR (pY1173) were found for the high-risk group of the pan-SCC cohort (Figure S23B,C). Significantly higher MEK1 (pS217/221) phosphorylation levels were also detected for the high-risk group of HPV16-negative tumors of the pan-SCC cohort, and two SCC subtypes (TCGA-HNSC and TCGA-CESC), while a similar trend was also evident for TCGA-LUSC and TCGA-ESCA, which did not reach statistical significance (Figure S24A,B).

To further validate accelerated MEK1 signaling as a characteristic feature and potential drug target for high-risk SCCs, 30 cases of the HIPO-HNC cohort (GSE117973) [20] were stratified into high-risk and low-risk groups (Figure S25A,B) based on transcript levels of the 18 gene signature, and stained by IHC. Prominent staining for phospho-MEK1/2 (pS217/221) was detected in cancer cells of most high-risk HNSCCs (11 out of 15 cases) but was only detected in four out of 15 cases for the low-risk HNSCCs at different subsites (Figure 6F).

## 3. Discussion

SCCs represent the most frequent human solid tumors and are a major cause of cancer mortality. They share common histological features, epidemiological risk factors, as well as molecular patterns [9,11]. Previous studies focused on the tumor immune microenvironment [13,14,15], biological and genetic traits [9], as well as oncogenic pathways [11], while others unrevealed germline variants as prognosticators in the pan-cancer landscape [27,28,29]. However, the poor 5-year survival rate in distinct HNSCC and other SCC subtypes [3] highlighted the urgent need for a better prognostic stratification and understanding of the underlying molecular principles to establish a more effective and less toxic therapy of SCCs at a higher risk for treatment failure and tumor recurrence. Hence, we describe a new risk stratification model for SCCs with a distinct clinical outcome based on a survival-related gene signature, which was established in a pan-SCC training cohort of TCGA and confirmed in independent HNSCC validation cohorts. Most relevant prognostic genes were prioritized by a LASSO Cox regression model and were used to identify subgroups with high or low-risks for unfavorable survival. An integrative analysis of multi-omics data among low-risk and high-risk groups highlighted FN1, SEMA3A, CDH2, FBN1, COL5A1, and ADAM12 as key nodes of a regulatory network, and finally predicted MEK-ERK and JAK-STAT3 signaling as promising drug targets for high-risk SCC patients. Presented data could pave the way for a better prognostic stratification of HNSCC and other SCC patients with an unfavorable clinical outcome, who may benefit from a more efficient and personalized treatment in future clinical trials.

In the first part of this study, 37 survival-related candidate genes were identified of which twelve candidates were differentially expressed among matched tumor and normal tissue. It is worth noting that only half of these candidate genes shared the expected up-regulation in tumor tissue and higher expression in SCC with an unfavorable prognosis or vice versa. This finding is of particular relevance as many previous studies reporting prognostic gene signatures focused on DEGs among tumors and normal tissues [30,31]. Our data indicate a certain risk for missing clinically relevant prognostic genes by focusing on DEGs among normal and tumor tissue, which is supported by An et al. [32].

Subsequently, 18 survival-relevant candidate genes were identified based on a LASSO Cox regression model applied on a large pan-SCC cohort. Several candidate genes of this signature, in particular candidate genes related to unfavorable survival, are already well-known prognostic factors. Previous studies reported that the up-regulation of CTSL or PTX expression correlates with poor prognosis [33] as well as lymph node or distant metastases, tumor stage, and overall survival in HNSCC [34,35,36,37]. Moreover, ITGA5 was identified as a candidate for partial EMT [24], and was related to unfavorable prognosis in HNSCC [38,39]. Regulation of SLC16A3 by DNA methylation and its prognostic value was reported in a previous pan-cancer study, including ESCA, HNSC, and LUSC [40]. A similar regulation or prognostic value was demonstrated in pancreatic cancer [41], lung adenocarcinoma [42], and clear cell renal cell carcinoma [43,44]. TMEM92 was reported in a 14 gene signature related to unfavorable survival of LUSC [45]. Finally, BZW1 and CAMK2A were not reported in SCC tumors yet; however, they were identified as prognostic markers in non-SCC tumors [46,47,48,49]. In the past, several prognostic biomarkers or gene sets have been published of which some have been confirmed by systematic reviews and meta-analyses for HNSCC more recently [50,51,52]. It is worth noting that none of them are included in our list of survival-related candidate genes and consequently are not part of the prognostic 18 gene signature for risk prediction. Confirmation of the prognostic 18 gene signature by a meta-analysis will generate a higher level of clinical evidence and remains a major challenge for future studies but is currently hampered by limited accessibility of publicly available datasets, including RNA-seq and survival data from independent pan-SCC cohorts.

A somatic mutation analysis elucidated a higher frequency of NSD1 and PIK3CA mutations in the low-risk group of the pan-SCC cohort, but also in all analyzed SCC subtypes. In 2017, Peri et al. reported a favorable survival of HNSCC with NSD1 mutations accompanied by global DNA hypomethylation [53], which is also a characteristic feature of low-risk SCCs in our study. As a histone methyltransferase, a previous study indicated that NSD1 mutations alter the methylation of histone H3 at K36 (H3K36), subsequently blocking cellular differentiation and promoting oncogenesis in HPV-negative HNSCC [22]. Other studies demonstrated that NSD1-mediated H3K36me2 is required for the recruitment of DNMT3A and maintenance of DNA methylation at intergenic regions in mouse cells [54], and that NSD1 mutations define a low immune infiltration phenotype in several SCCs [55,56].

By applying an integrative analysis of multi-omics data taking into account both genetic and epigenetic events and their impact on DEGs among low-risk and high-risk groups of the pan-SCC cohort, we unraveled FN1, SEMA3A, CDH2, FBN1, COL5A1, and ADAM12 as key nodes of a gene regulatory network. Interestingly, FN1 and CDH2 were well established markers for epithelial-to-mesenchymal transition (EMT) and promoted metastasis in several SCCs [56,57,58,59]. Furthermore, FN1 is a glycoprotein of the extracellular matrix, which enables interactions between tumor cells and the extracellular matrix and plays essential roles in cell adhesion and dissemination processes [60,61].

Furthermore, two MEK inhibitors (Trametinib and Selumetinib) were predicted as effective compounds for high-risk SCCs by an in-silico drug screen. This prediction was further supported by a higher activity of the MAPK-ERK pathway in high-risk as compared with low-risk SCCs. The well-established oncogenic role of the MEK-ERK pathway [62,63,64,65] has made this pathway a primary drug target for numerous cancers [66,67]. The efficacy of MEK inhibitors as monotherapy or in combinatorial treatment strategies was supported by several pre-clinical or clinical trials in several primary or metastatic settings [68,69,70,71,72,73]. Interestingly, a higher frequency of RASA1 mutations was found in the high-risk SCC cohort and cell lines, which is a negative regulator of the RAF–MEK–ERK pathway. RASA1 can enhance the intrinsic GTPase activity of RAS, resulting in an increase of inactive GDP-bound forms of Ras, thereby leading to an aberrant intracellular signaling through the RAF–MEK–ERK pathway [74]. On the other hand, more frequent KRAS mutations were evident in the high-risk SCC cohort and cell lines, which play an essential role in controlling the activity of multiple downstream effectors, including MEK-ERK signaling [75]. Aberrant RAS function is closely associated with a single mutation, typically at codon 12, 13, or 61. Mutations at these conserved sites favor GTP binding and lead to constitutive activation of the RAS–MEK–ERK pathway [76], which is in line with more KRAS mutations and enrichment of its activity in high-risk SCC. Though our study demonstrated a higher staining pattern for phospho-MEK1/2 in cancer cells of HNSCC with a higher risk pattern, a limitation of the presented data is the small sample size and confirming in larger cohorts, including SCC from other anatomical sites, is a major challenge in future studies.

It is also worth noting that Ngan et al. reported a favorable survival of HNSCC patients with somatic mutations in key components of the MAPK pathway [77], which raises concerns on the clinical benefit of targeted inhibition of the MEK-ERK pathway as a “magic bullet” for all SCC. However, tumors might exhibit accelerated MEK-ERK pathway activity despite missing MAPK pathway mutations due to the activation of upstream regulators (e.g., EGFR) by genomic alterations or high levels of stimulating ligands [78]. This assumption is in line with computational inferred pathway activities, which are not necessarily associated with the mutational landscape of key components in the respective pathways as demonstrated recently for EGFR or PI3K pathways [78,79]. On the other hand, Ngan et al. provide compelling experimental evidence that HNSCC with MAPK pathway mutations share an immune-active tumor microenvironment with a CD8-positive T cell-inflamed phenotype [77]. In contrast, numerous studies reported an association between EGFR-MEK pathway activity with an immune cold phenotype in multiple cancers, including HNSCC, and a lower benefit from ICI therapy in cancers with EGFR mutations [80]. These data underline the urgent need for a better cellular and molecular stratification of high-risk SCC patients, who might benefit from the targeted inhibition of the MEK-ERK pathway.

Similar to other integrative analyses of multi-omics data derived from bulk tumor tissue, this study shares some limitations. For example, the LASSO Cox model established on RNA sequencing data has limited power using datasets based on microarray platforms. Moreover, the risk model should be confirmed by a prospective analysis of a large SCC patient cohort, and the efficacy of MEK inhibition for high-risk SCC requires validation in appropriate pre-clinical models and future clinical trials.

## 4. Methods and Methods

### 4.1. Expression and Clinical Datasets Acquisition

Messenger RNA, functional miRNAs, and lncRNA expression data were downloaded for 1466 tumors across TCGA-CESC (*n* = 304, including 252 SCC and 52 non-SCC), TCGA-ESCA (*n* = 161, including 81 SCC and 80 non-SCC), TCGA-HNSC (*n* = 500), and TCGA-LUSC (*n* = 501) cohorts from https://portal.gdc.cancer.gov/ (accessed on 8 November 2019) and protein expression levels were downloaded from https://www.tcpaportal.org/tcpa (accessed on 15 November 2019). In total, RNA-seq data were available for 1334 SCC for further analysis. The pan-cancer clinical and follow-up data were accessed from the https://gdc.cancer.gov/ in 28 November 2019. Information on the HPV status was accessed from Cao et al. [81].

Transcriptome data for HNSCC validation cohorts were downloaded from Gene Expression Omnibus (GSE65858 [19] and GSE41613 [21]) in 18 January 2021 or were available from HIPO-HNC (GSE117973 [20]). For GSE65858 (*n* = 270), we considered only primary HNSCC (*n* = 253) for further analysis.

Transcriptome and drug screening data of the Cancer Cell Line Encyclopedia (CCLE) cell lines were downloaded from cBioPortal (https://www.cbioportal.org/) [82,83,84] in 18 February 2020. We selected SCC cell lines (*n* = 66) from the cervix, oesophagus, upper aerodigestive tract, and lung for further analysis.

### 4.2. Survival Analyses

The best cutoff for OS or PFI of distinct SCC cohorts, respectively, was computed by “maxstat” (smethod = “LogRank”, pmethod = “exactGauss”, and abseps = 0.01) in R. OS, DSS, and PFI probabilities were calculated for a combined cohort or individual cohorts using the Kaplan–Meier method and the log-rank test was used to compare the differences among groups. The survival analysis and visualization were performed by R packages “survminer”, “survival” and “ggplot2”. A univariable Cox regression analysis was performed by the R package “survival”, and the hazard ratio and 95% confidence interval were computed.

### 4.3. Consensus Clustering

Consensus clustering was performed by “ConsensusClusterPlus” package in R, with 80% cases resampling, k-means clustering algorithm upon 1-spearman correlation distances, and seed as 123456. SCC tumors were classified from 2 to 10 clusters and the partition was determined by the consensus cumulative distribution function evaluating the consensus matrix [85].

### 4.4. Random Forest Regression

Random forest regression was performed by the “randomForest” (seed = 100) and visualized by the “ggplot2” package in R.

### 4.5. The LASSO Cox Regression

The LASSO Cox regression was used for prioritizing the most relevant prognostic candidate genes by the “glmnet” package in R. The prioritized gene set was used to establish the regression risk model based on the pan-SCC cohort of TCGA [85,86]. The risk score was computed by the R package “glmnet” (s = lambda.min and type = “response”), and the analytical formula for risk assessment was derived on the basis of 18 prioritized candidate genes (coefficients of genes: RPS6KA5 = −0.091314273; EVA1C = −0.028241615; FOXRED2 = −0.015921459; ITPRIPL1 = −0.015085757; TIAM1 = −0.009605322; FAM83C = −0.001895369; NOS2 = −0.001432844; BZW1 = 0.000472265; CTSL = 0.00075636; TPRG1 = 0.001472244; ITGA5 = 0.001809248; SLC16A3 = 0.005035462; PTX3 = 0.005254341; CAMK2A = 0.007942951; SERINC3 = 0.010006911; SUSD1 = 0.020496547; EDA2R = 0.025931937 and TMEM92 = 0.043746806).

The same risk score cut-off was applied to all cases of the pan-SCC cohort from TCGA for stratification into the low- or high-risk groups.

### 4.6. ESTIMATE Immune Score

Stromal, immune, and ESTIMATE scores for distinct SCC cohorts were computed by the R package “estimate” [87].

### 4.7. Somatic Mutation Analysis

The mutation counts and candidate genes with a MutSig 2.0 q-value < 0.05 for TCGA-CESC, TCGA-ESCA, TCGA-HNSC, and TCGA-LUSC cohorts were accessed from cBioPortal in 23 January 2020. Significant enrichment among distinct prognostic clusters were analyzed by a Chi-square test. The somatic mutation data of CCLE cell lines were accessed from cBioPortal in 18 February 2020.

### 4.8. CNV Analysis

The CNA fractions of individual SCC cohorts from TCGA were downloaded from cBioPortal in 9 January 2020. The CNA fraction data of CCLE cell lines were downloaded from cBioPortal in 18 February 2020.

Global CNA data (Level_3_segmented_scna_minus_germline_cnv_hg19_seg) of TCGA-CESC, TCGA-ESCA, TCGA-HNSC, and TCGA-LUSC cohorts were downloaded from http://www.firebrowse.org/ in 29 January 2020. The segment mean < −0.2 was defined as loss and >0.2 as gain. CoNVaQ web tool (https://convaq.compbio.sdu.dk/, accessed on 9 March 2020) [88] was used as a statistical model for distinct prognostic clusters based on Fisher’s exact test. CNA plots were visualized with IGV_2.4.19 (Integrative Genomics Viewer_2.4.19) [89].

### 4.9. Differential Expression Analysis

Differentially expressed gene, miR, and lncRNA analyses were performed by the “EdgeR” package in R [90].

### 4.10. DNA Methylation Analysis

DNA methylation data (Methylation 450k) of TCGA-CESC, TCGA-ESCA, TCGA-HNSC, and TCGA-LUSC cohorts were downloaded from https://gdc.xenahubs.net/ in 19 February 2020. Methylation 450k data were normalized with the R package “limma”. The global methylation value was computed as beta mean values of probes annotated for gene promoters (*n* = 13,564) with the most variable beta values among the top 30,000 probes with the highest beta mean value. Significant differences among distinct prognostic clusters were computed by the Wilcoxon test.

The processed methylation data of CCLE cell lines were downloaded from Gene Expression Omnibus (GSE68379) [91] in 18 February 2020.

### 4.11. Target Gene Prediction

The MiRcode database [92] was used for differentially expressed lncRNA to establish an miR link prediction on highly conserved miR families. Three online databases (TargetScan V7.2 [93], miRDB V6.0 [94,95], and mirTarBaseV7.0 [96]) were used for functional miR-DEG link prediction. The visualization of the network structure was completed by Cytoscape [97].

### 4.12. Genome Annotation and Enrichment Analyses

The STRING database (Search Tool for Recurring Instances of Neighboring Genes) [98] was used to establish the network structure for multi-omics regulated candidate genes. The STITCH database (Search Tool for Interacting Chemicals) [99] was used for establishing the protein–chemical interaction networks.

Kyoto Encyclopedia of Genes and Genomes (KEGG) and GO hypergeometric enrichment analyses were processed by the R package “clusterProfiler” [100]. The Gene Set Enrichment Analysis (GSEA) algorithm was used to compute the normalized enrichment score and statistical significance for Molecular Signatures Database (MSigDB) hallmark, C2, C5, as well as C6 collection terms and gene set permutations were performed 1000 times for each analysis by GSEA v4.0.3 software.

### 4.13. Single Cell Sequencing Analysis

The Cell Browser online database (https://cells.ucsc.edu/, accessed on 19 June 2020) was used to analyze single cell sequencing data from ten HNSC patients (GSE103322) [24].

### 4.14. Single Sample Gene Set Enrichment Analysis (ssGSEA)

Enrichment scores were computed by ssGSEA applying the “GSVA” package in R [101]. The lists of ST_ERK1_ERK2_MAPK_PATHWAY and HALLMARK_IL6_JAK_STAT3_SIGNALING signatures were accessed from https://www.gsea-msigdb.org/gsea (accessed on 9 July 2020).

### 4.15. Immunohistochemistry (IHC) Staining

IHC staining was performed as described previously (19) on formalin-fixed paraffin-embedded (FFPE) tumor sections of the HIPO-HNC cohort with a rabbit anti-phospho-MEK1/2(Ser217/221) antibody (#9121, Cell Signaling Technology). The specificity of the staining was confirmed with a rabbit IgG isotype control antibody (DA1E, Cell Signaling Technology) (data not shown). FFPE tumor sections were provided by the tissue bank of the National Center for Tumor Disease (Institute of Pathology, University Hospital Heidelberg, Heidelberg, Germany).

### 4.16. Study Approval

Patients of the HIPO-HNC cohort (GSE117973) were treated between 2012 and 2016 at the University Hospital Heidelberg, Germany. Patient samples were obtained and analyzed under protocols S-206/2011 and S-220/2016, approved by the Ethics Committee of Heidelberg University, with written informed consent from all participants. This study was conducted in accordance with the Declaration of Helsinki.

## 5. Conclusions

In conclusion, we established a prognostic risk model for pan-SCC and identified potential drug targets and predicted effective compounds. Our data pave the way for innovative pre-clinical studies and future clinical trials stratified for SCC patients at higher risk for treatment failure and tumor relapse.

## Data Availability

The datasets generated and/or analyzed during the current study are available from the corresponding author on reasonable request. Expression profile data of TCGA cohorts from https://portal.gdc.cancer.gov/ (accessed on 8 November 2019) and protein expression levels were downloaded from https://www.tcpaportal.org/tcpa (accessed on 15 November 2019). The TCGA pan-cancer clinical and follow-up data were accessed from the https://gdc.cancer.gov/ (accessed on 28 November 2019) and HPV information was accessed from the publication of Cao et al. (DOI: 10.1038/srep28294). Transcriptome data for the validation cohort with 81 primary HNSC were available from HIPO-HNC (GSE117973) (DOI: 10.1002/ijc.32481). Transcriptome and drug screening data of the CCLE cell lines were downloaded from cBioPortal (https://www.cbioportal.org/, accessed on 18 February 2020).

## Supplementary Materials

The following are available online at https://www.mdpi.com/article/10.3390/cancers13205182/s1, Figure S1: Establishment of a prognostic gene signature for the pan-SCC cohort, Figure S2: GO enrichment analysis for survival-related candidates of the pan-SCC cohort, Figure S3: Expression of survival-related candidate genes (*n* = 37) among matched tumor and normal tissues of the pan-SCC, HNSC or LUSC cohorts, Figure S4: Consensus matrix for survival-related candidate genes of the pan-SCC cohort derived from consensus clustering analysis, Figure S5: Subgroup analysis for the prognostic pan-SCC classifier, Figure S6: Consensus clustering analysis for pan-SCC survival-related candidate genes in GSE117973, GSE65858 and GSE41613 validation cohorts, Figure S7: Survival analyses for a combined HNSCC validation cohort, Figure S8: LASSO Cox regression model for the pan-SCC cohort based on survival-related candidate genes (*n* = 37), Figure S9: Subgroup analysis for low-risk versus high-risk cases of the pan-SCC cohort, Figure S10: HPV16 and HPV18-related subgroup analysis for low-risk versus high-risk cases of CESC and HNSC cohorts, Figure S11: Association between risk groups and ESTIMATE signatures for individual TCGA cohorts, Figure S12: Differences in somatic mutations among high-risk and low-risk groups of individual TCGA cohorts, Figure S13: Subgroup analysis for global CNA fraction, Figure S14: Differences in genomic CNA among high-risk and low-risk groups of individual TCGA cohorts, Figure S15: Subgroup analysis for global DNA methylation, Figure S16: Subgroup analysis for differentially methylated probe signatures, Figure S17: The lncRNA-miR-DEG network structure, Figure S18: Single cell sequencing analysis for HNSC, Figure S19: Risk stratification of SCC cell lines based on the 18-gene signature, Figure S20: Differences in the mutational landscape and DNA methylome among SCC cell lines with a high or low risk score, Figure S21: MAPK/ERK pathway activity in SCC cell lines and subgroups of the pan-SCC cohort, Figure S22: JAK-STAT3 pathway activity in SCC cell lines and subgroups of the pan-SCC cohort, Figure S23: EGFR-RAF-MEK pathway analysis of the pan-SCC cohort according to the TCPA dataset, Figure S24: Subgroup analysis for MEK1 phosphorylation, Figure S25: Risk stratification of the GSE117973 cohort, Table S1: Summary of prognostic-related candidate genes, Table S2: Summary of the survival-related 37-gene set, Table S3: DEGs among low-risk and high-risk groups of the pan-SCC cohort, Table S4: Significant MSigDB hallmark terms among low-risk versus high-risk groups of the pan-SCC cohort by GSEA, Table S5: Significant MSigDB C2 (curated gene sets) terms among low-risk versus high-risk groups of the pan-SCC cohort by GSEA, Table S6: Significant MSigDB C5 (GO gene sets) terms among low-risk and high-risk groups of the pan-SCC cohort by GSEA, Table S7: Significant MSigDB C6 (oncogenic gene sets) terms among low-risk versus high-risk groups of the pan-SCC cohort by GSEA, Table S8: DEGs affected by copy number alteration among low-risk versus high-risk groups of the pan-SCC cohort, Table S9: DEGs affected by promoter methylation among low-risk versus high-risk groups of the pan-SCC cohort, Table S10: Differentially expressed miRs low-risk versus high-risk groups of the pan-SCC cohort, Table S11: Differentially expressed lncRNAs among low-risk versus high-risk groups of the pan-SCC cohort, Table S12: Candidate genes affected in at least three out of four genetic or epigenetic models.
