# Supplementary material for: Prognostic Gene Signature for Squamous Cell Carcinoma with a Higher Risk for Treatment Failure and Accelerated MEK-ERK Pathway Activity
Source: Cancers (Basel). 2021 Oct 15;13(20):5182. doi: 10.3390/cancers13205182 (PMC8534038; doi:10.3390/cancers13205182)
Supplement: Supplementary file 1 [file cancers-13-05182-s001.zip › cancers-1380615 supplementary figures.pdf]

Supplementary Figures

Prognostic Gene Signature for Squamous Cell Carcinoma with a Higher Risk for Treatment Failure and Accelerated MEK-ERK Pathway Activity

Bohai Feng, Kai Wang, Esther Herpel, Michaela Plath, Wilko Weichert, Kolja Freier, Karim Zaoui and Jochen Hess

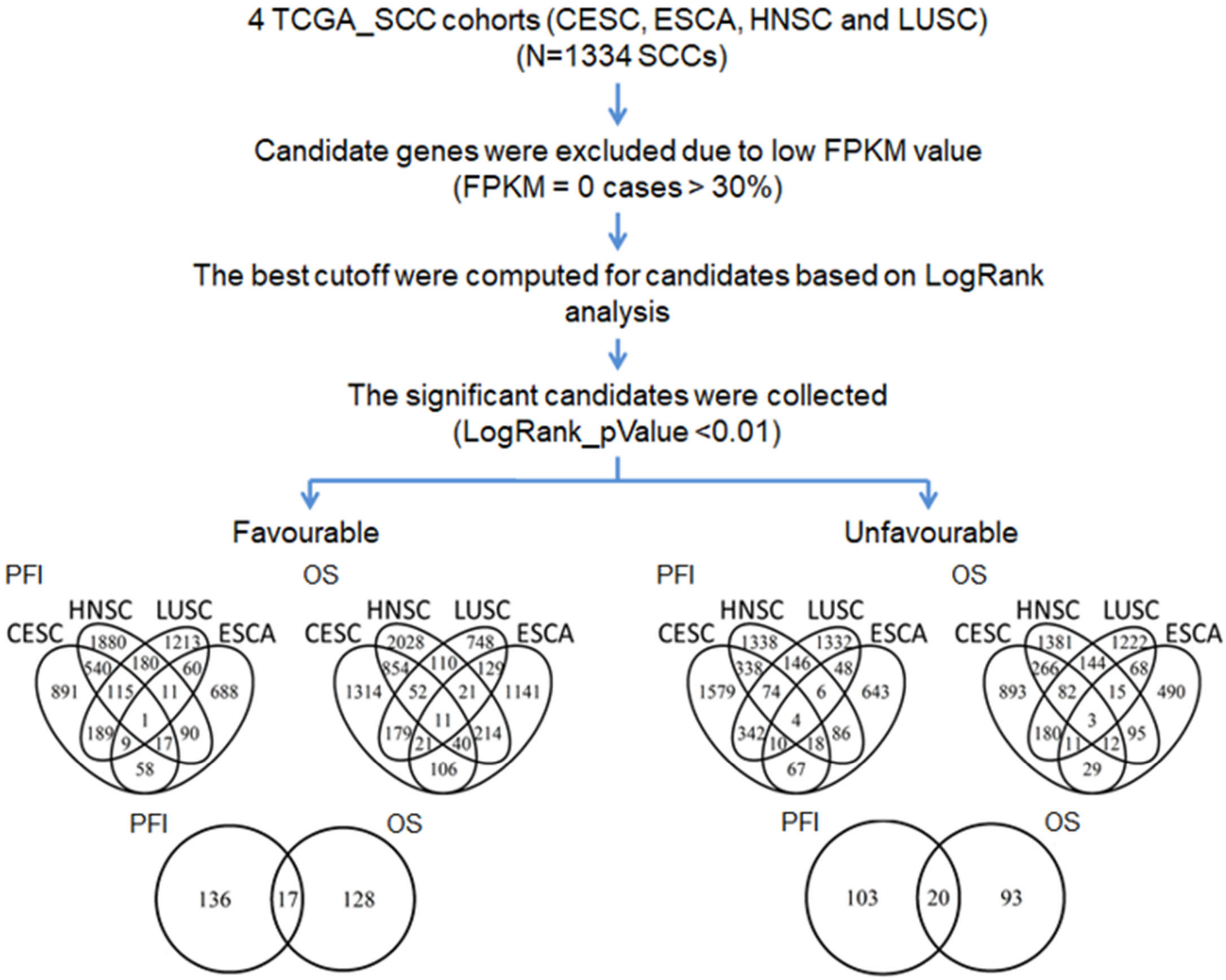

Figure S1. Establishment of a prognostic gene signature for the pan-SCC cohort. Schematic summary of the workflow to compute survival-related candidate genes (n = 37) for the pan-SCC cohort, which consisted of four independent TCGA cohorts (CESC, ESCA, HNSC, LUSC). Venn diagrams illustrate amount of candidate genes related to significant differences in overall survival (OS) or progression-free intervals (PFI) for individual cohorts (top), or for selected candidate genes with significant differences in at least three out of four cohorts (bottom).

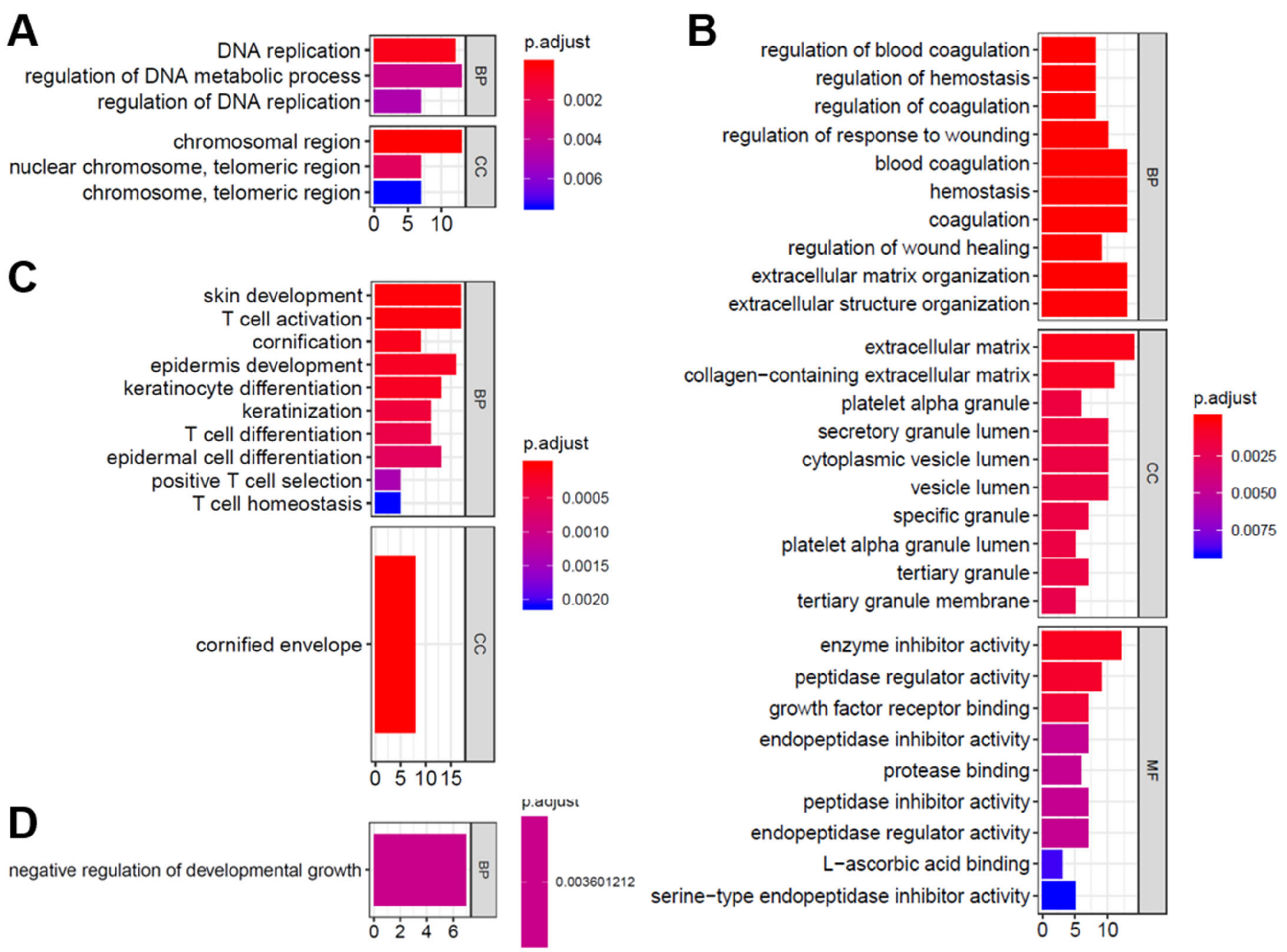

Figure S2. GO enrichment analysis for survival-related candidates of the pan-SCC cohort. Graphs present top Scheme 145. or an unfavorable OS (B; n = 113), and candidate genes related to a favorable PFI (C; n = 153) or an unfavorable PFI (D; n = 123) according to GO enrichment analysis.

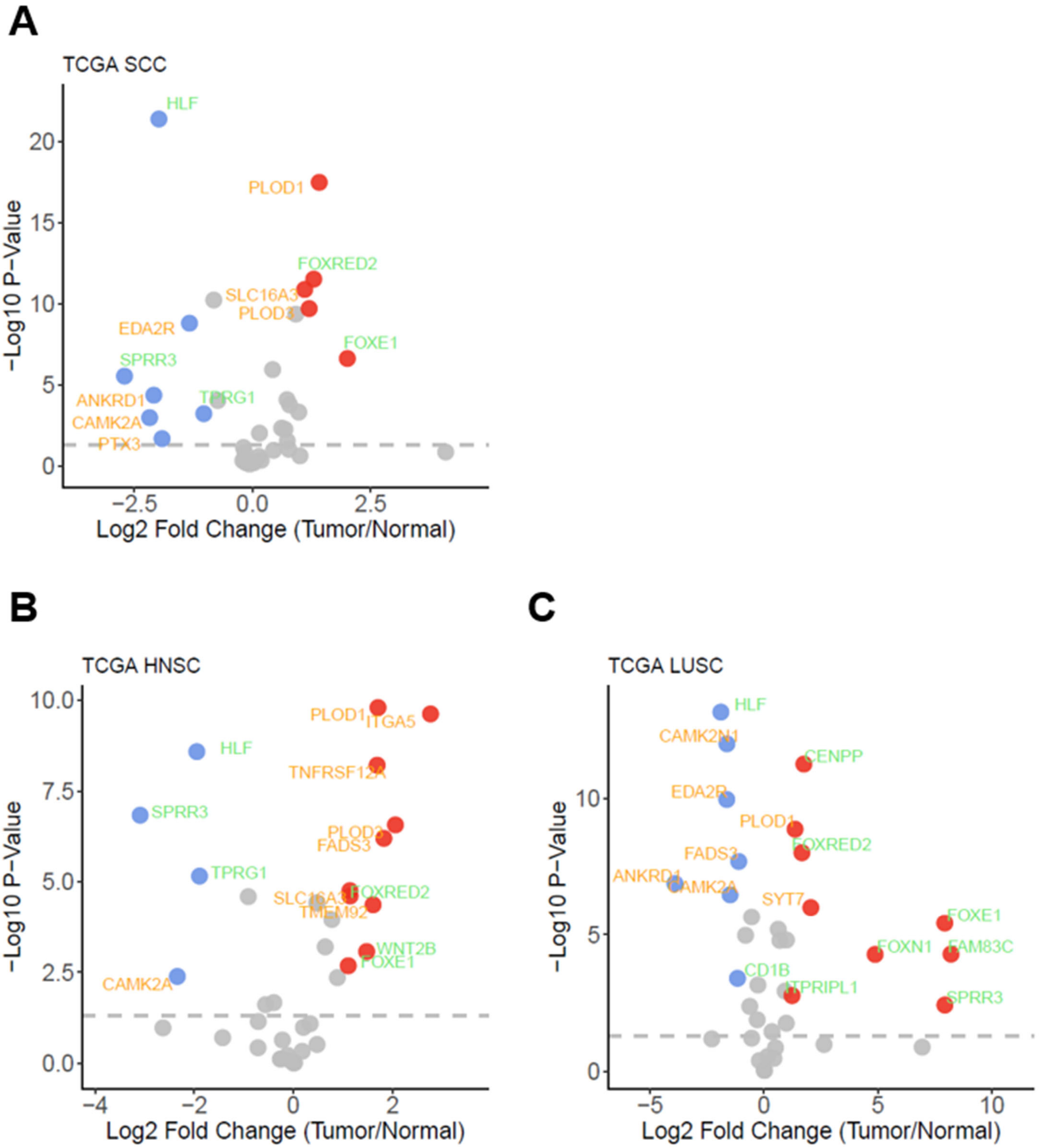

Figure S3. Expression of survival-related candidate genes (n = 37) among matched tumor and normal tissues of the pan-SCC, HNSC or LUSC cohorts. Volcano plots present differentially expression genes (−1 > log2FC > 1, *P* value < 0.05) of the survival-related candidate gene set (n = 37) among matched tumor and normal tissues of the pan-SCC (**A**), HNSC (**B**) and LUSC (**C**) cohorts. Red dots mean the DEGs up regulated in tumor, and blue dots mean the DEGs up regulated in normal tissue. The color of gene symbols means the directions of the survival-related candidate genes (favorable survival in green and unfavorable survival in orange).

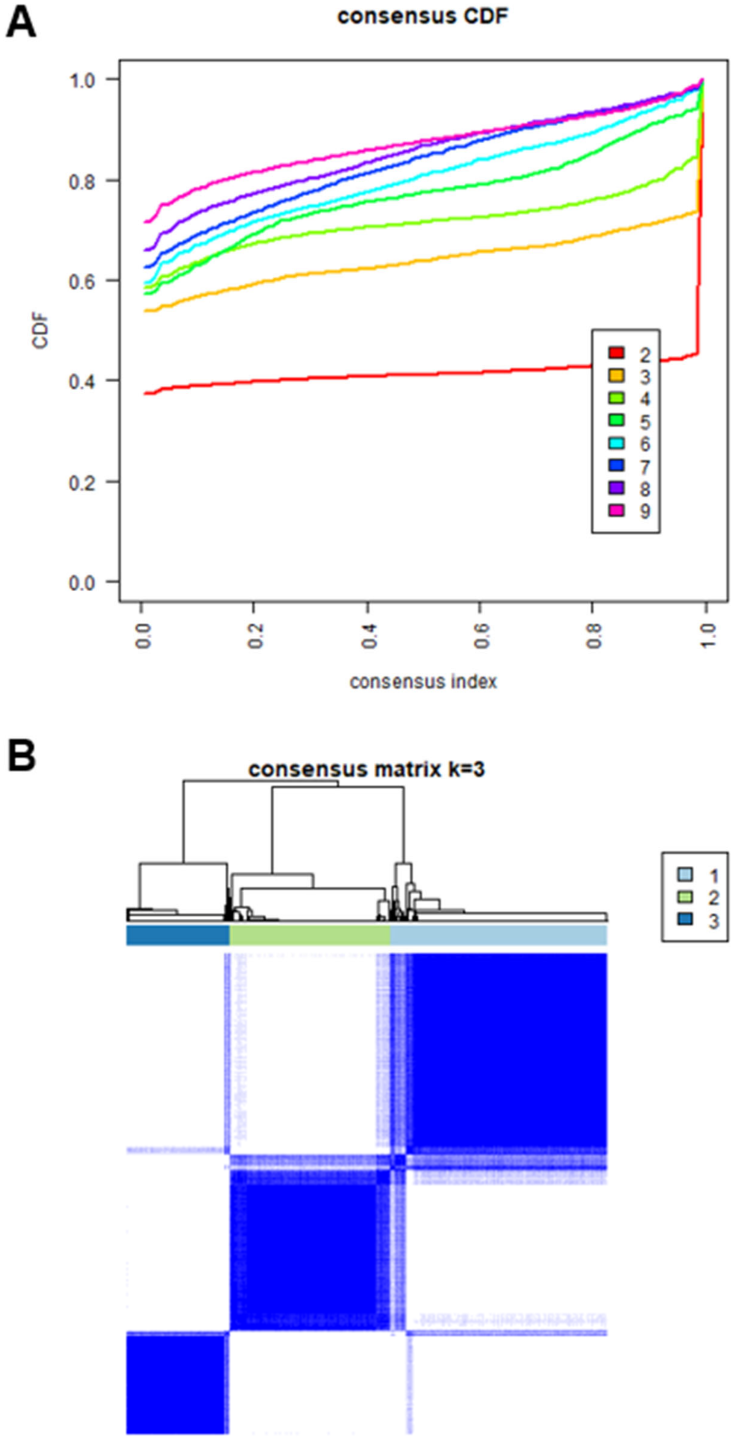

Figure S4. Consensus matrix for survival-related candidate genes of the pan-SCC cohort derived from consensus clustering analysis. (**A**) Consensus clustering of the cumulative distribution function (CDF) for k = 2 to 9. (**B**) The color-map represents the probability that the pan-SCC cohort was clustered across 50 sampling runs based on consensus clustering.

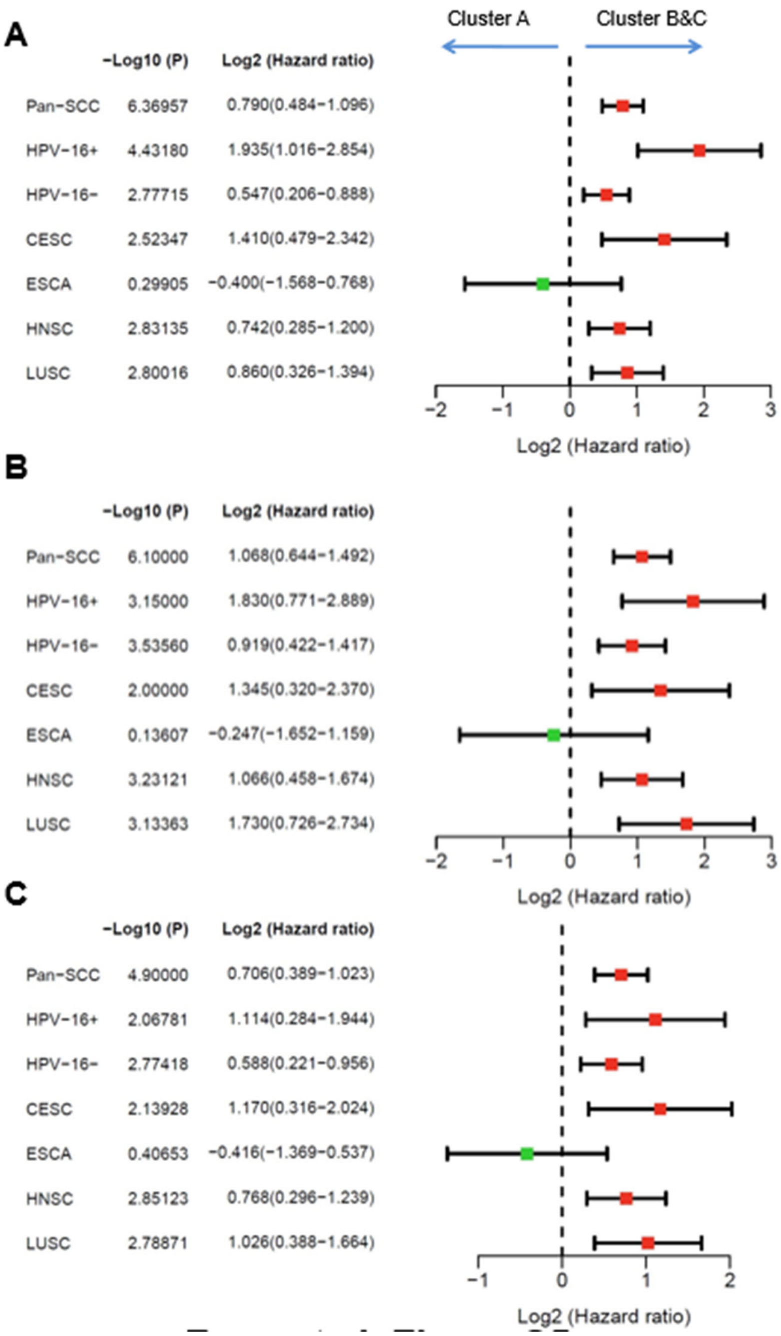

Figure S5. Subgroup analysis for the prognostic pan-SCC classifier. Forest plots summarize hazard ratios (HR) and 95% CI for the association between clusters (cluster A vs. cluster B&C) and 5-year OS (**A**), DSS (**B**) and PFI (**C**) based on following strata: pan-SCC, HPV16 status (positive or negative), and tumor type (CESC, ESCA, HNSC and LUSC). Green squares = 0 < HR < 1; red squares = HR > 1.

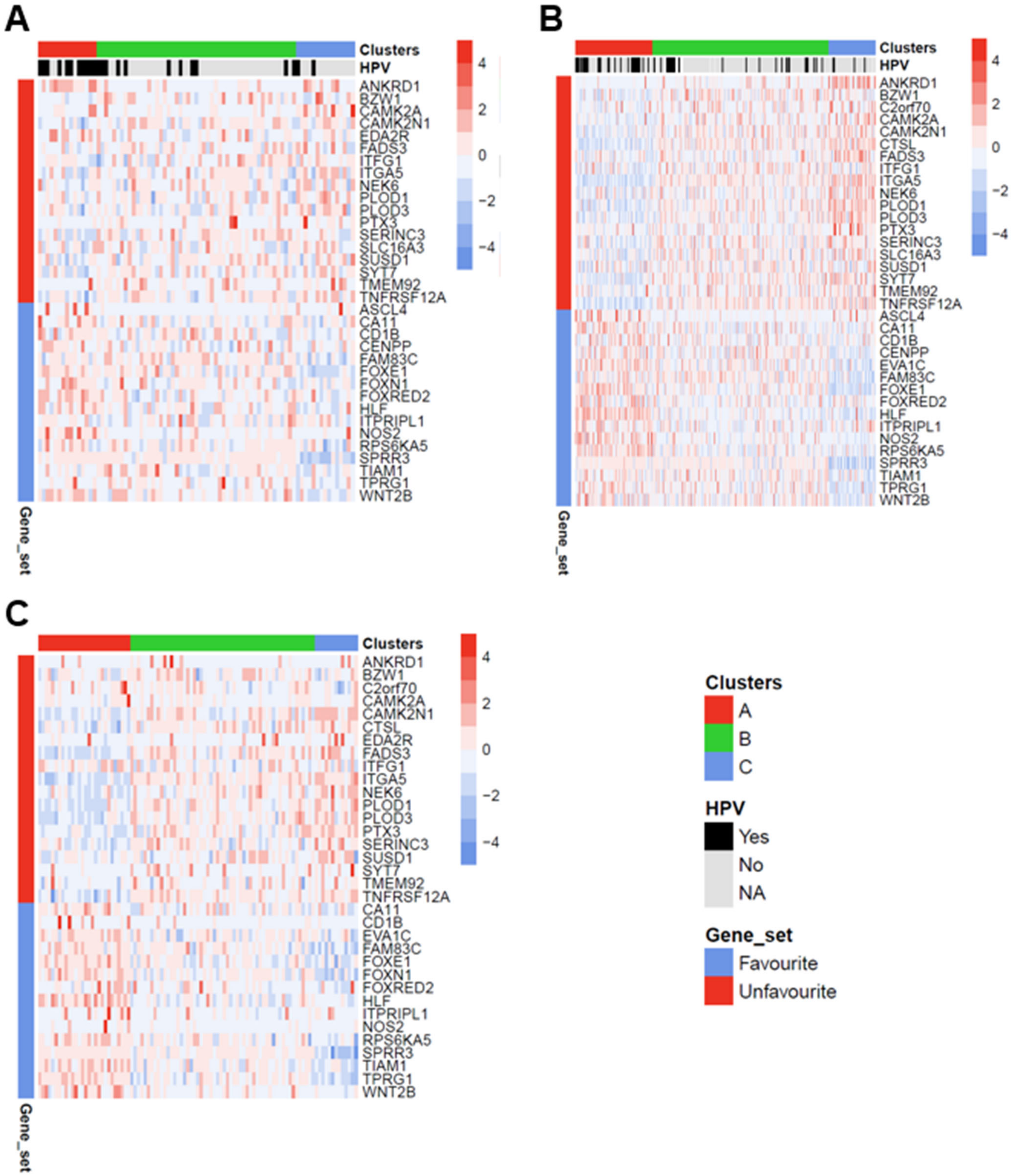

Figure S6. Consensus clustering analysis for pan-SCC survival-related candidate genes in GSE117973, GSE65858 and GSE41613 validation cohorts. Consensus clustering analysis confirmed distinct sub-clusters based on pan-SCC survival-related candidate gene signature in GSE117973 (**A**), GSE65858 (**B**) and GSE41613 (**C**) validation cohorts.

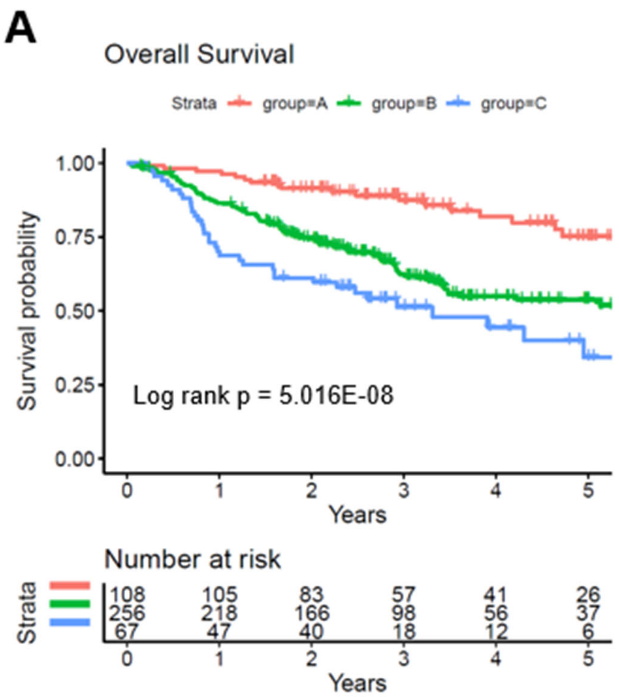

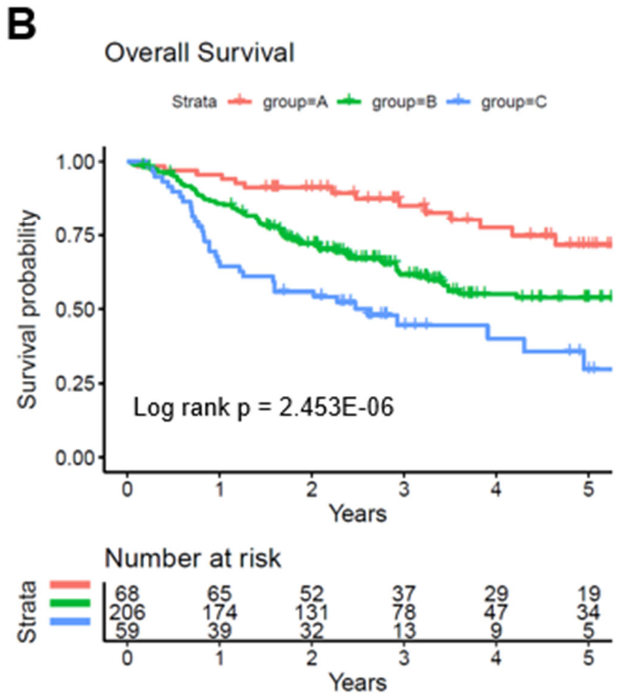

Figure S7. Survival analyses for a combined HNSCC validation cohort. Kaplan-Meier plots display 5-year OS differences between cluster A, B and C in a combined HNSCC validation cohort (GSE117973, GSE65858 and GSE41613) (**A**), and in the HPV-negative subgroup (**B**). Log-rank statistic was conducted to test for statistical significance.

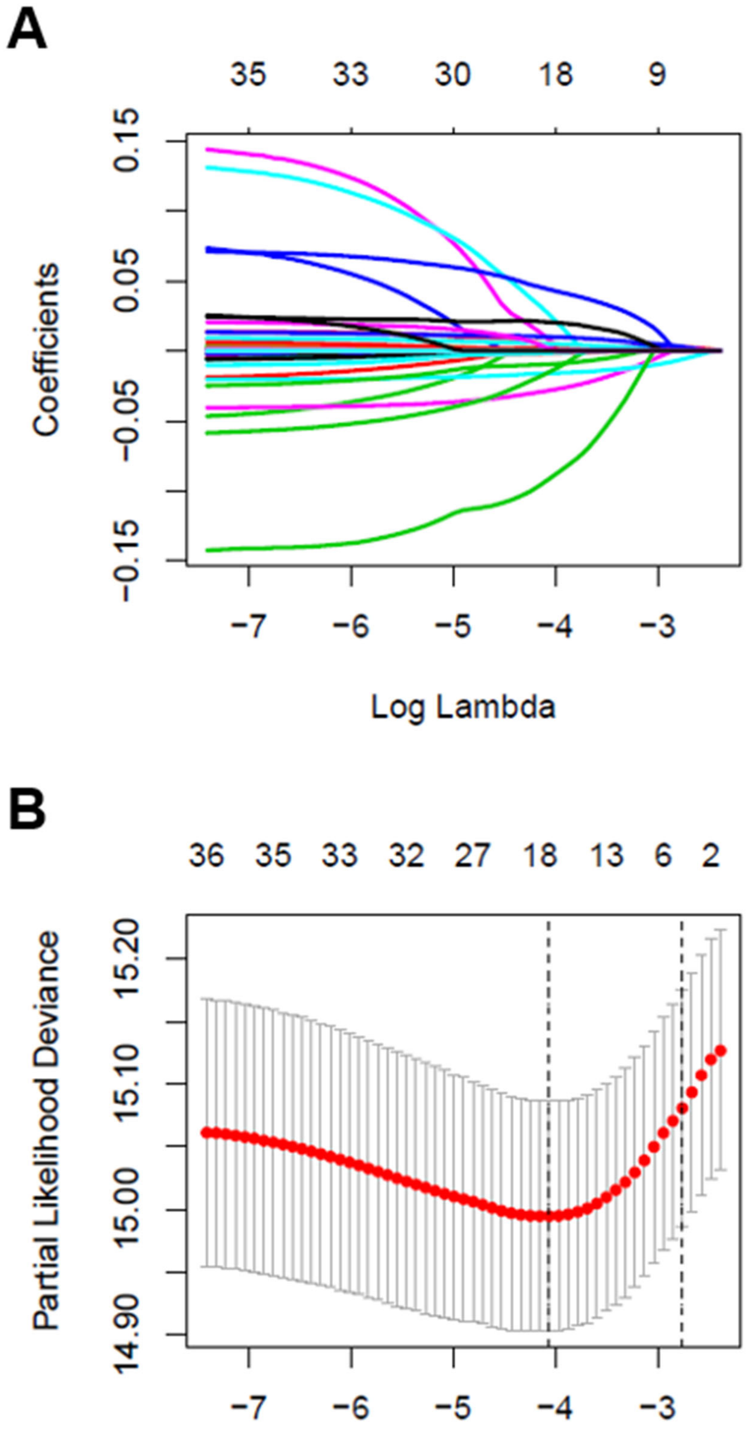

Figure S8. LASSO Cox regression model for the pan-SCC cohort based on survival-related candidate genes (*n* = 37). (**A**,**B**) The LASSO Cox regression model revealed 18 most relevant candidate genes for favorable or unfavorable OS of the pan-SCC cohort.

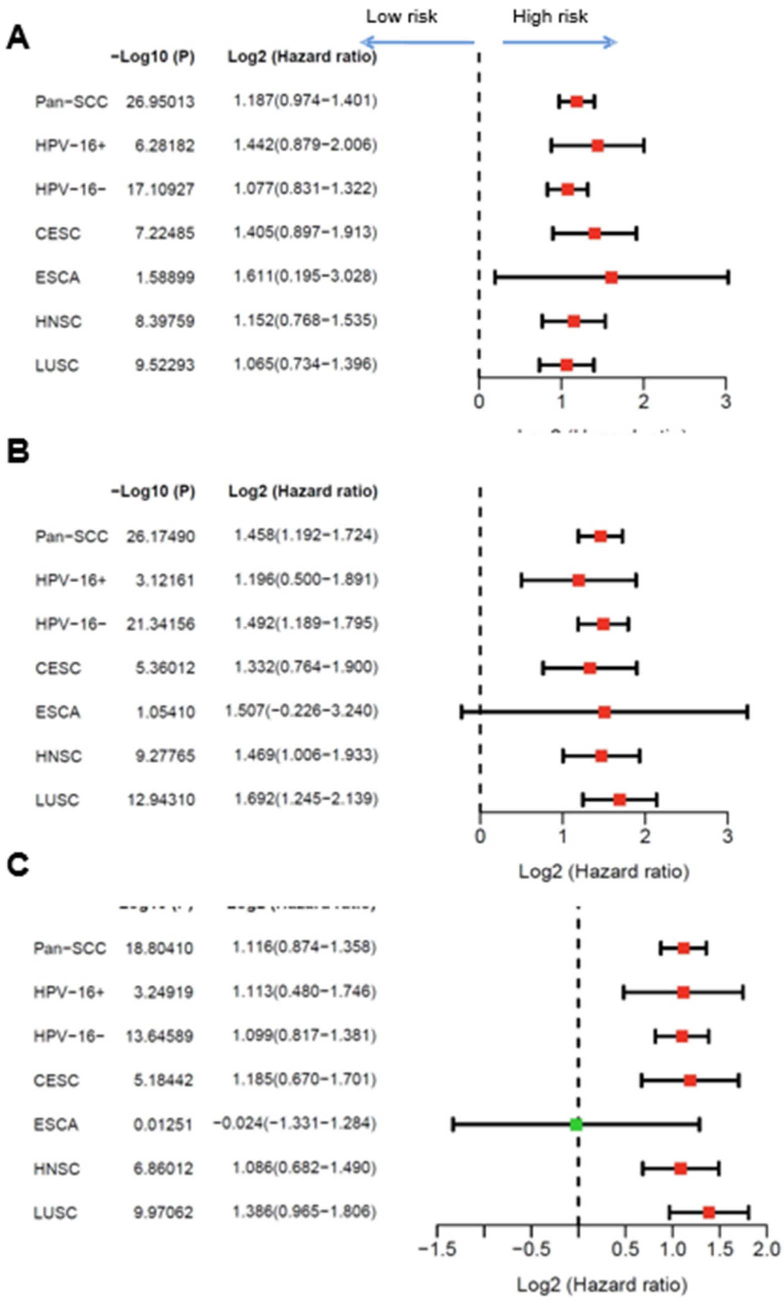

Figure S9. Subgroup analysis for low-risk versus high-risk cases of the pan-SCC cohort. Forest plots summarize hazard ratios (HR) and 95% CI for the association between risk groups (low-risk vs. high-risk) and 5-year OS (**A**), DSS (**B**) and PFI (**C**) based on following strata: pan-SCC, HPV16 status (positive or negative), and tumor type (CESC, ESCA, HNSC and LUSC). Green squares = 0 < HR < 1; red squares = HR > 1.

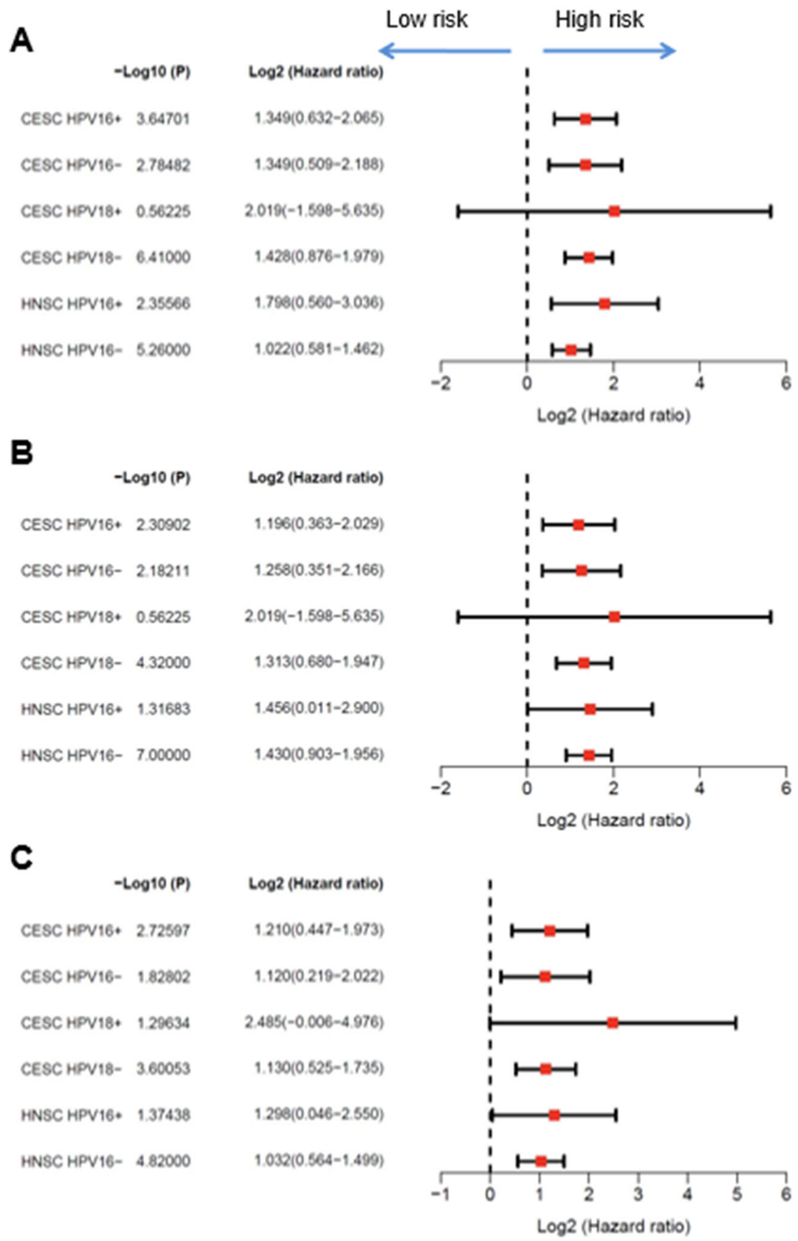

Figure S10. HPV16 and HPV18-related subgroup analysis for low-risk versus high-risk cases of CESC and HNSC cohorts. Forest plots summarize hazard ratios (HR) and 95% CI for the association between risk groups (low-risk vs. high-risk) and 5-year OS (A), DSS (B) and PFI (C) based on following strata: HPV16 and HPV18 status (positive or negative) in CESC as well as HPV16 status (positive or negative) in HNSC cohorts.

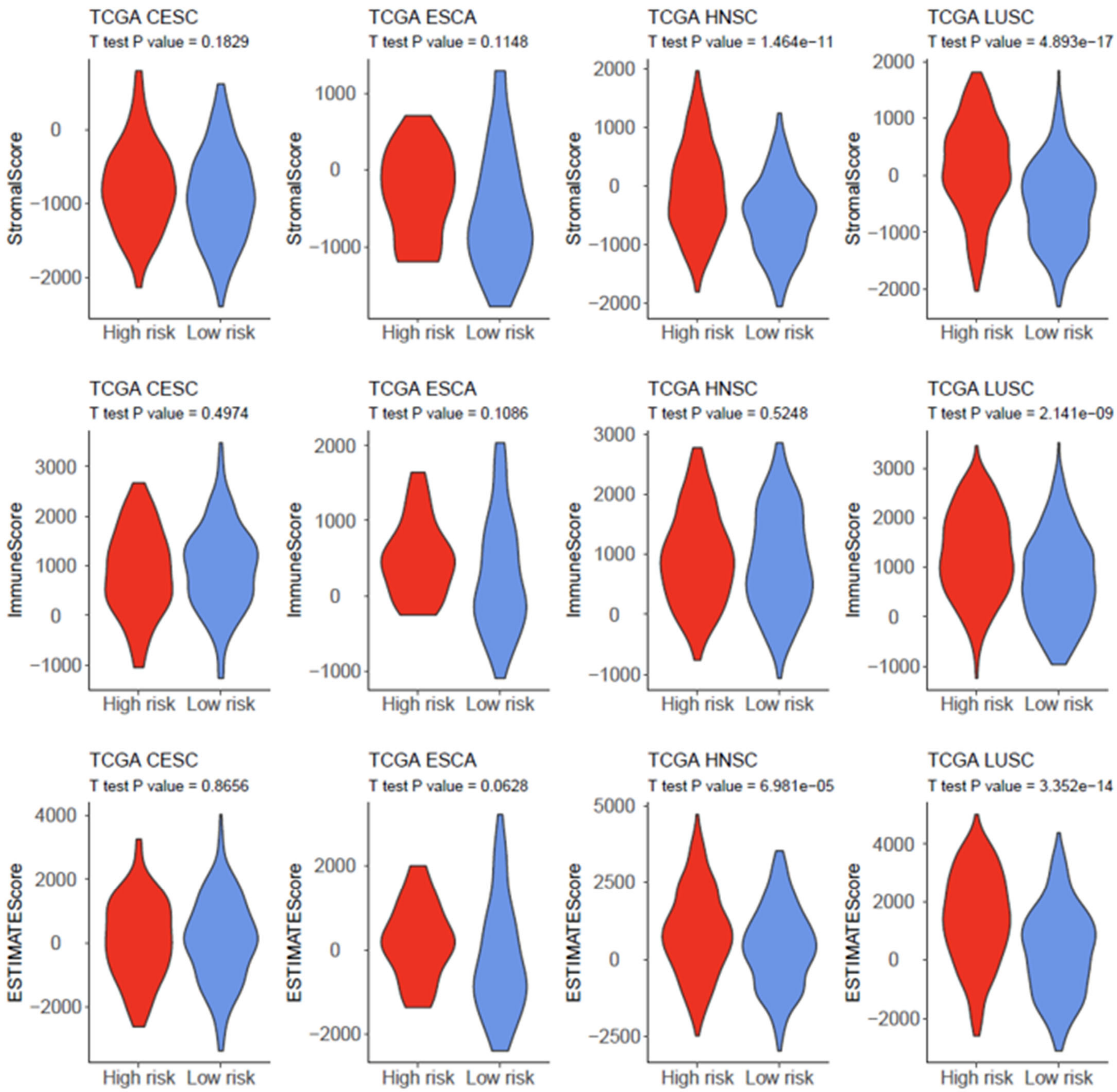

Figure S11. Association between risk groups and ESTIMATE signatures for individual TCGA cohorts. Violin plots illustrate distribution of stromal (upper), immune (middle), and ESTIMATE (lower) signature scores for low-risk and high-risk groups in indicated TCGA cohorts.

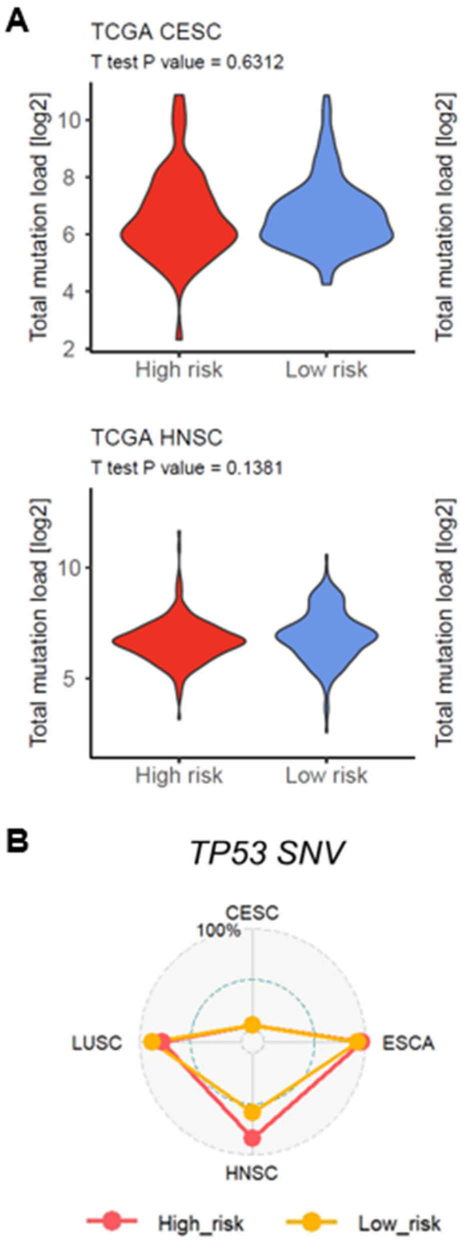

Figure S12. Differences in somatic mutations among high-risk and low-risk groups of individual TCGA cohorts. (**A**) Violin plots demonstrate no significant differences for total mutation load among high-risk and low-risk groups of indicated TCGA cohorts. (**B**) Graph shows relative somatic mutation frequencies for TP53 in high-risk or low-risk groups of indicated TCGA cohorts.

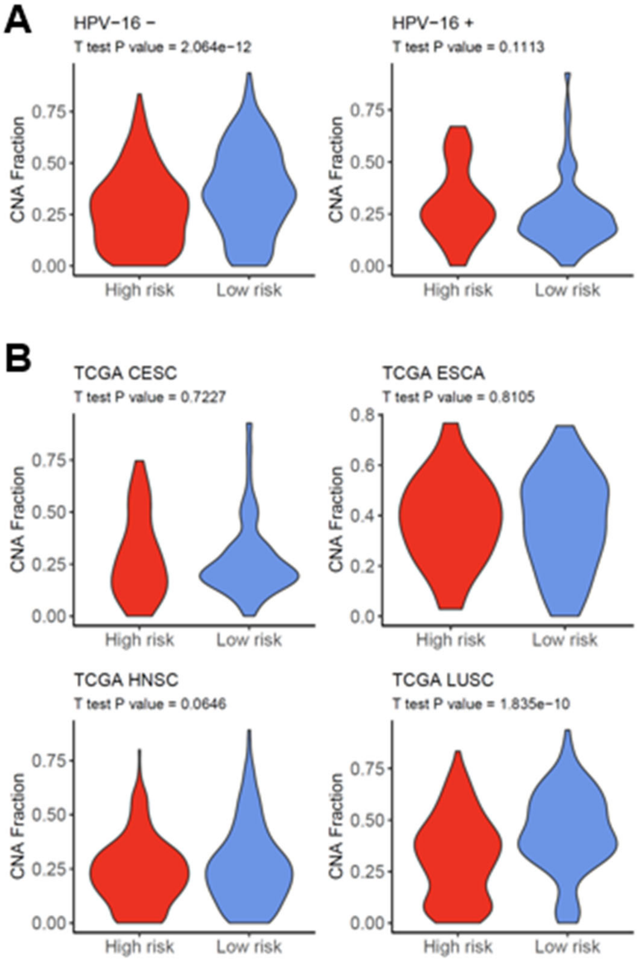

Figure S13. Subgroup analysis for global CNA fraction. (**A**) Violin plots demonstrate a significant higher level of the global CNA fraction for low-risk as compared to high-risk groups of HPV16-negative but not HPV16-positive SCCs. (**B**) Violin plots demonstrate the distribution of the global CNA fraction between high-risk and low-risk groups in indicated TCGA cohorts, which only reached statistical significance for TCGA-LUSC.

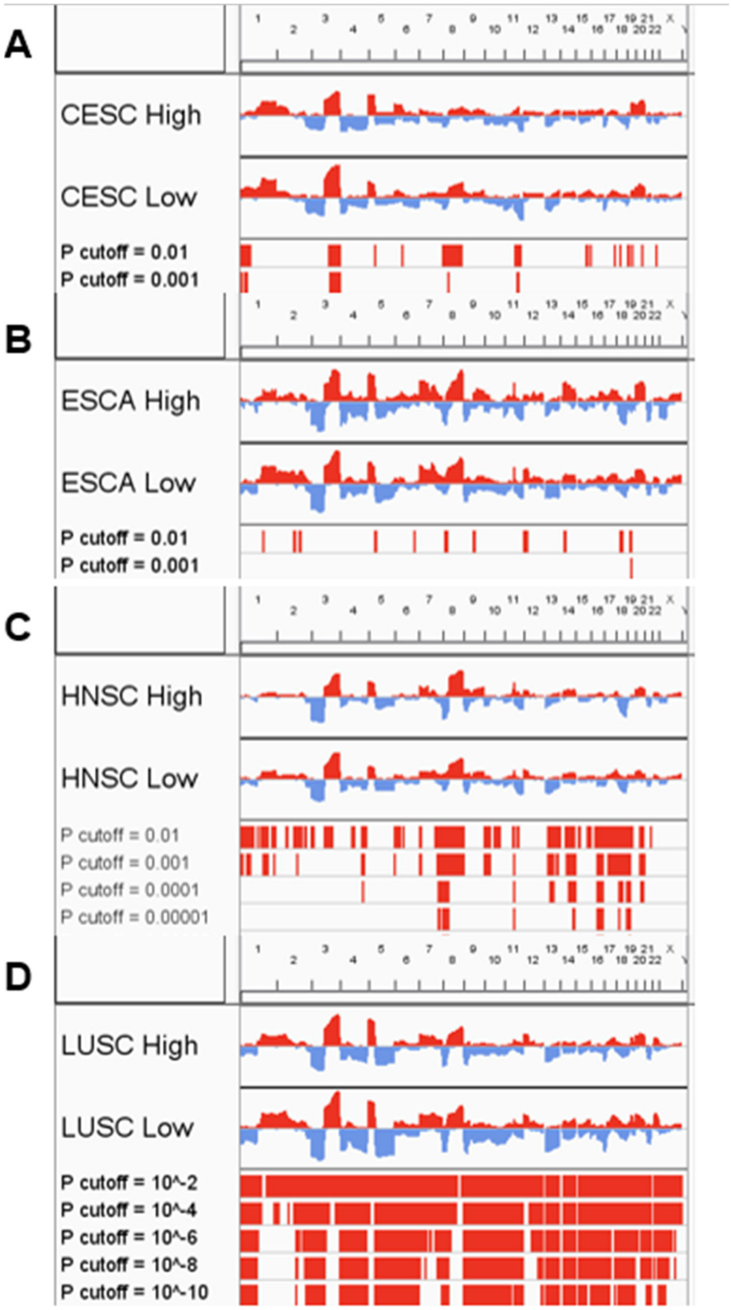

Figure S14. Differences in genomic CNA among high-risk and low-risk groups of individual TCGA cohorts. CNA plots show relative frequency of copy number gains (red) and deletions (blue) among high-risk or low-risk groups of indicated TCGA cohorts: TCGA-CESC (**A**), TCGA-ESCA (**B**), TCGA-HNSC (**C**), and TCGA-LUSC (**D**).

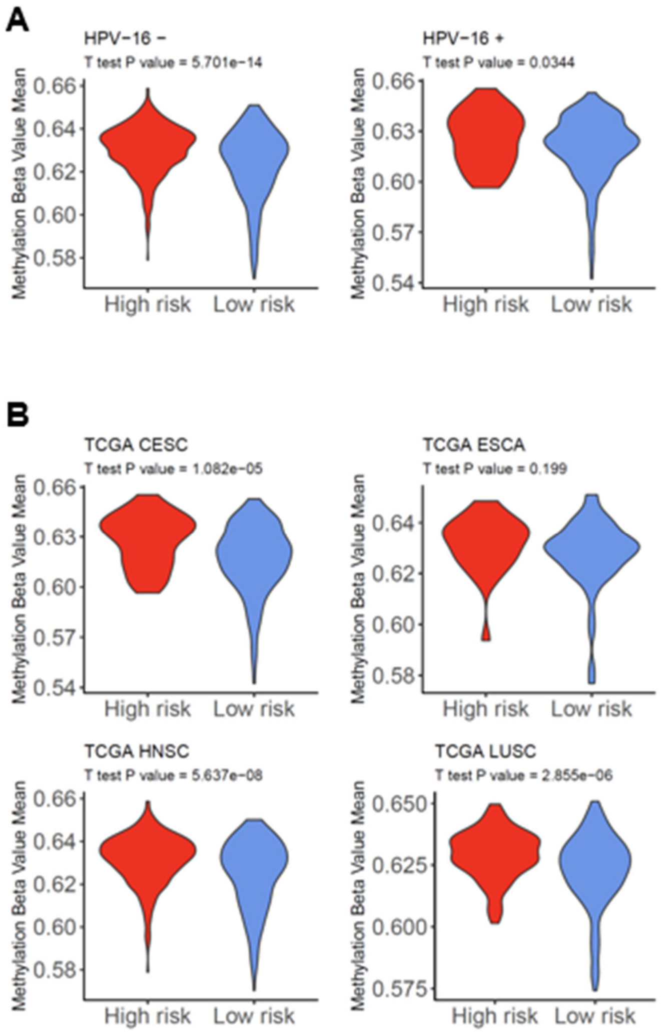

Figure S15. Subgroup analysis for global DNA methylation. (**A**) Violin plots demonstrate significant differences in global methylation values between high-risk and low-risk groups of HPV16-negative (left) and HPV16-positive (right) SCCs. (**B**) Violin plots demonstrate significant differences in global methylation values between high-risk and low-risk groups of indicated tumor entities, except for TCGA-ESCA.

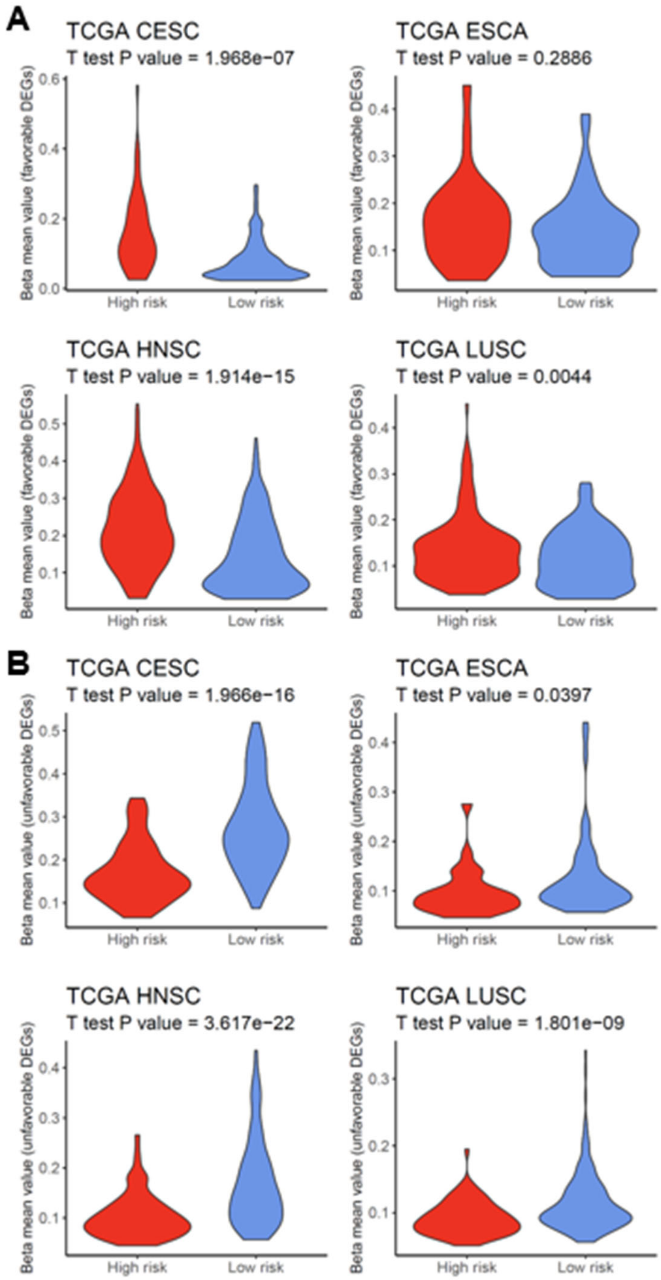

Figure S16. Subgroup analysis for differentially methylated probe signatures. Violin plots demonstrate significant differences in beta mean values of probe with higher methylation in the high-risk group (**A**) or low-risk group (**B**) for indicated tumor entities, except for TCGA-ESCA in (**A**).

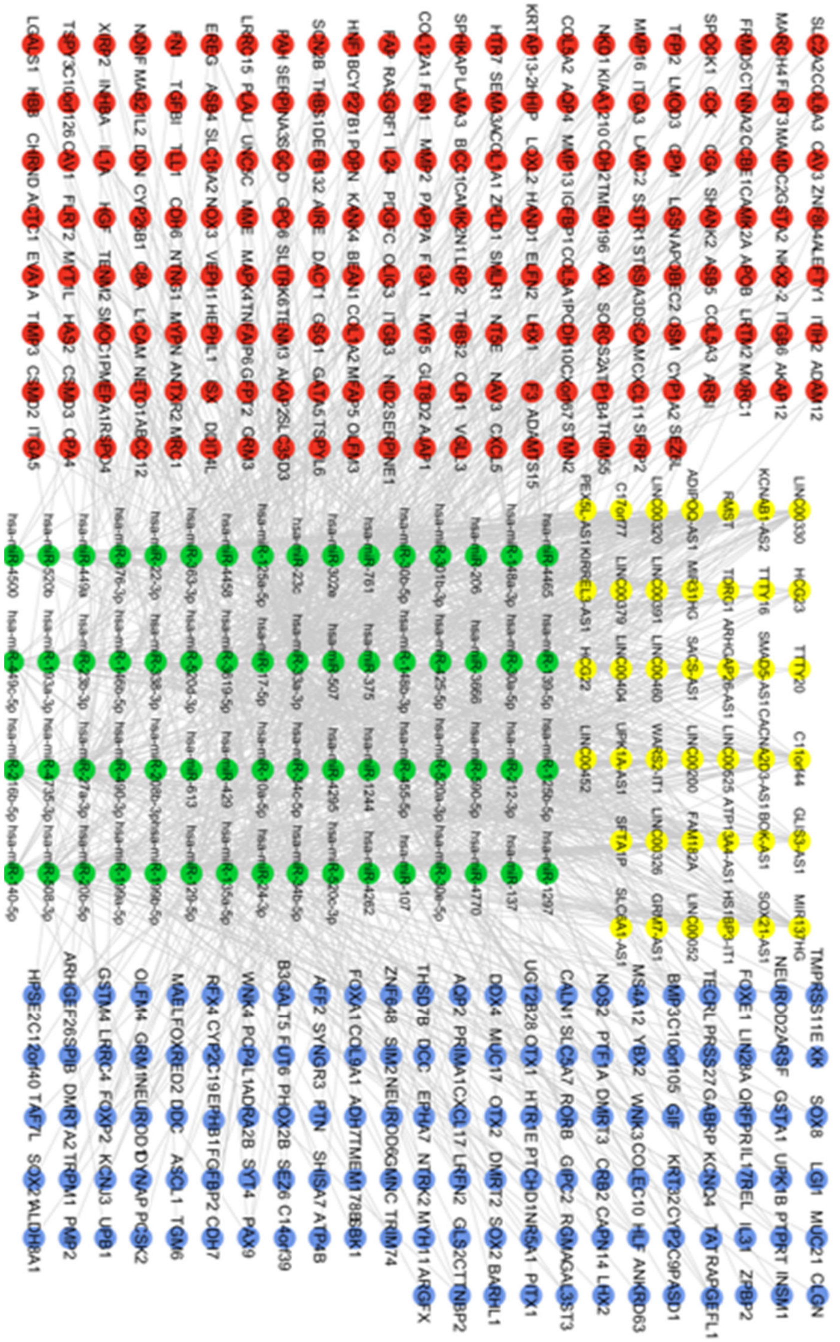

Figure S17. The lncRNA-miR-DEG network structure. The network summarizes complex connections between differentially expressed lncRNAs (yellow dots), lncRNA-targeted miRs (green dots), and DEGs (red dots = up regulated in the high-risk group; blue dots = up-regulated in low-risk group).

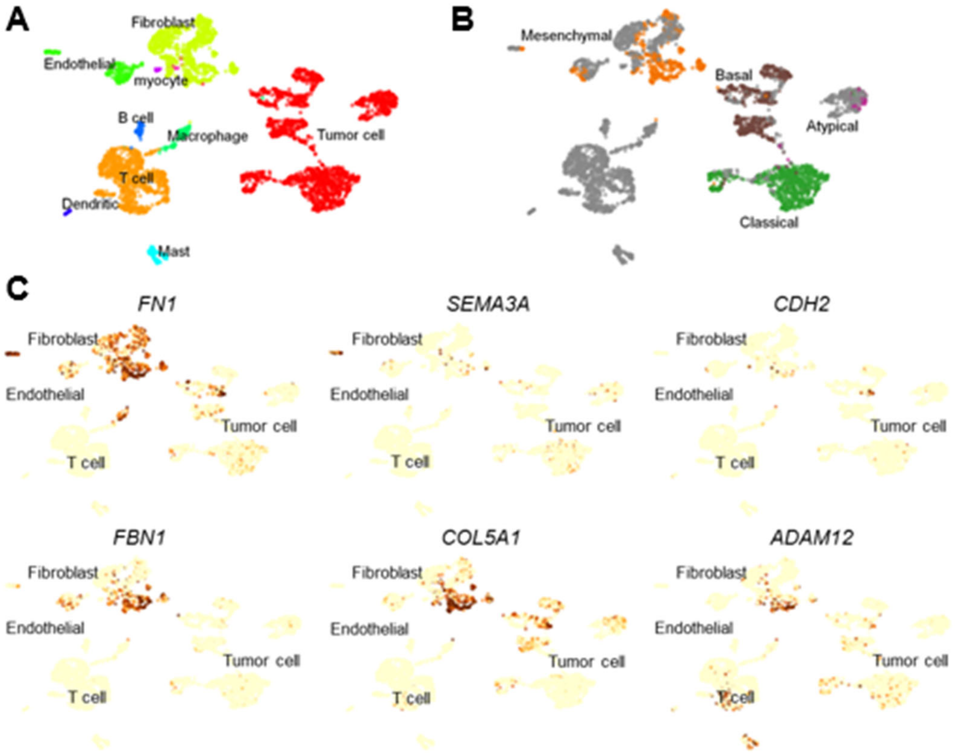

Figure S18. Single cell sequencing analysis for HNSC. (**A**) Uniform Manifold Approximation and Projection (UMAP) plot of cells from ten HNSC patients (GSE103322) reveals consistent clusters of stromal, immune and tumor cells across samples. Clusters are assigned to indicated cell types by differentially expressed genes. (**B**) UMAP plot colored by distinct TCGA expression subtypes (Green dots =Classical, Purple dots =Atypical, Brown dots =Basal, and Orange dots =Mesenchymal). (**C**) UMAP plots illustrates expression of selected candidate genes in indicated cell types from low (light brown) to high (dark brown).

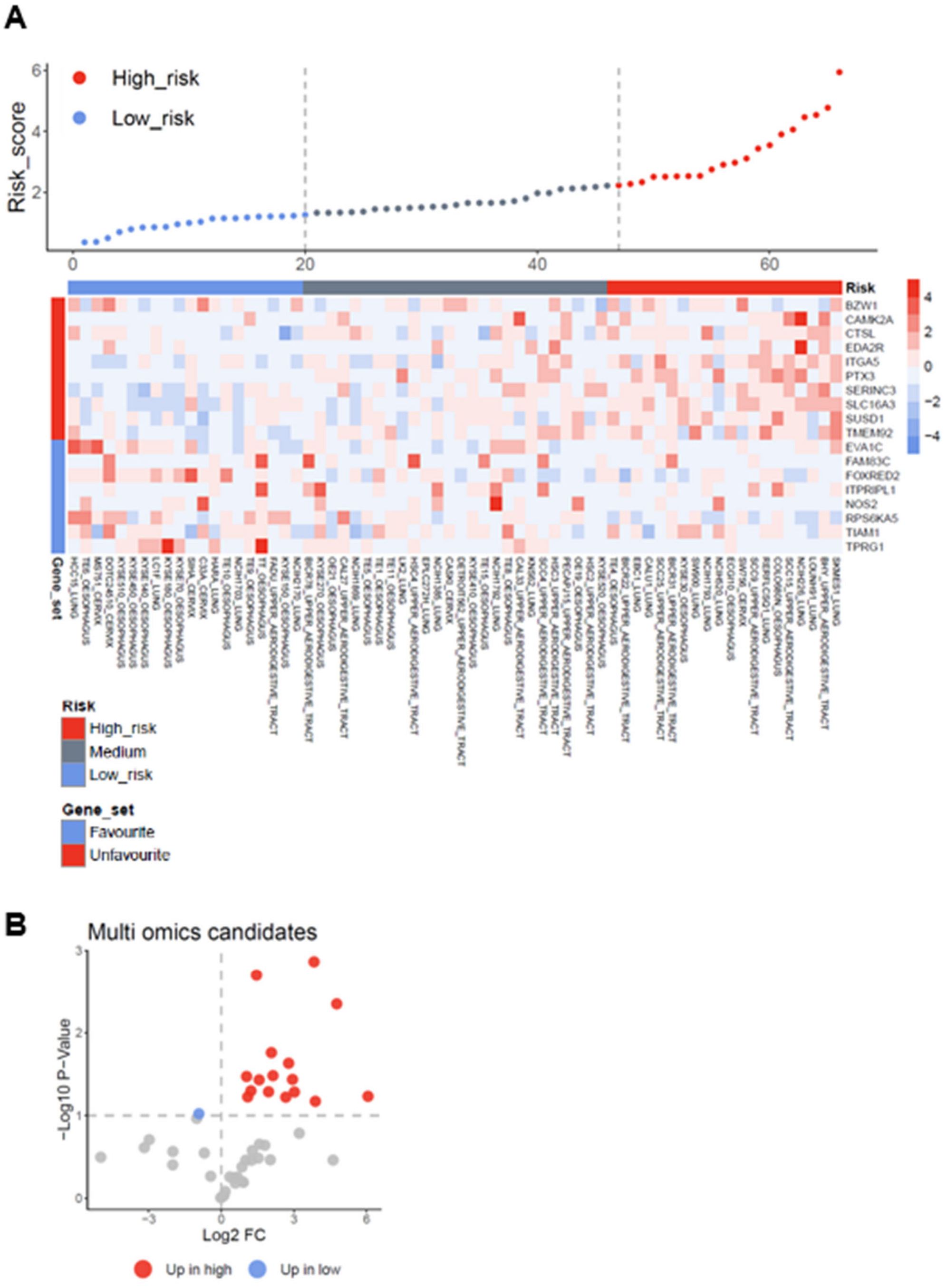

Figure S19. Risk stratification of SCC cell lines based on the 18-gene signature. (**A**) Dot plot displays SCC cell lines with a high (red dot), moderate (grey dot) or low (blue dots) risk score and the heatmap illustrates expression patterns of the survival-related 18-gene signature. (**B**) Volcano plot demonstrates differential expression of candidate genes among SCC cell lines with a high or low risk score with similar trend as compared to high-risk and low-risk groups of the pan-SCC cohort (cutoff $p = 0.1$).

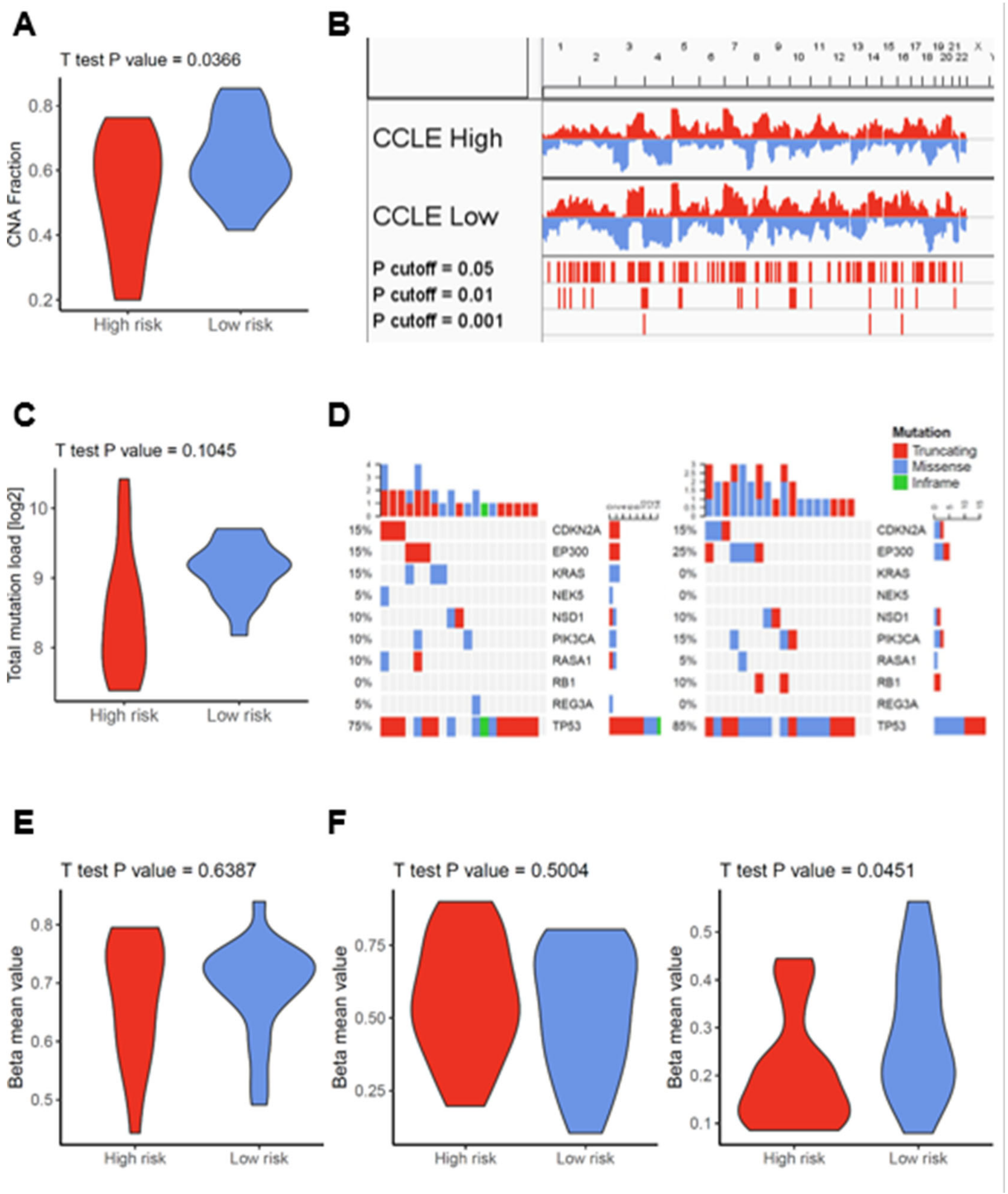

Figure S20. Differences in the mutational landscape and DNA methylome among SCC cell lines with a high or low risk score. (**A**) Violin plot demonstrates significant higher total CNA fraction in SCC cell lines with a lower as compared to a higher risk score. (**B**) CNA plots show relative frequencies of copy number gains (red) and deletions (blue) for SCC cell lines with a lower or a higher risk score. (**C**) Violin plot demonstrates no significant difference for total mutation load among SCC cell lines with a lower as compared to a higher risk score. (**D**) Oncomaps summarize frequencies of somatic mutations for SCC tumor significant MutSig genes (chi-square test, $p < 0.05$) in SCC cell lines with a lower as compared to a higher risk score. (**E**) Violin plot demonstrates a significantly higher beta mean value for global DNA methylation in SCC cell lines with a lower as compared to a higher risk score. (**F**) Violin plots demonstrate a significant difference for beta mean values of probes with higher expression in the low-risk groups of the pan-SCC cohort among SCC cell lines with a higher or lower risk score.

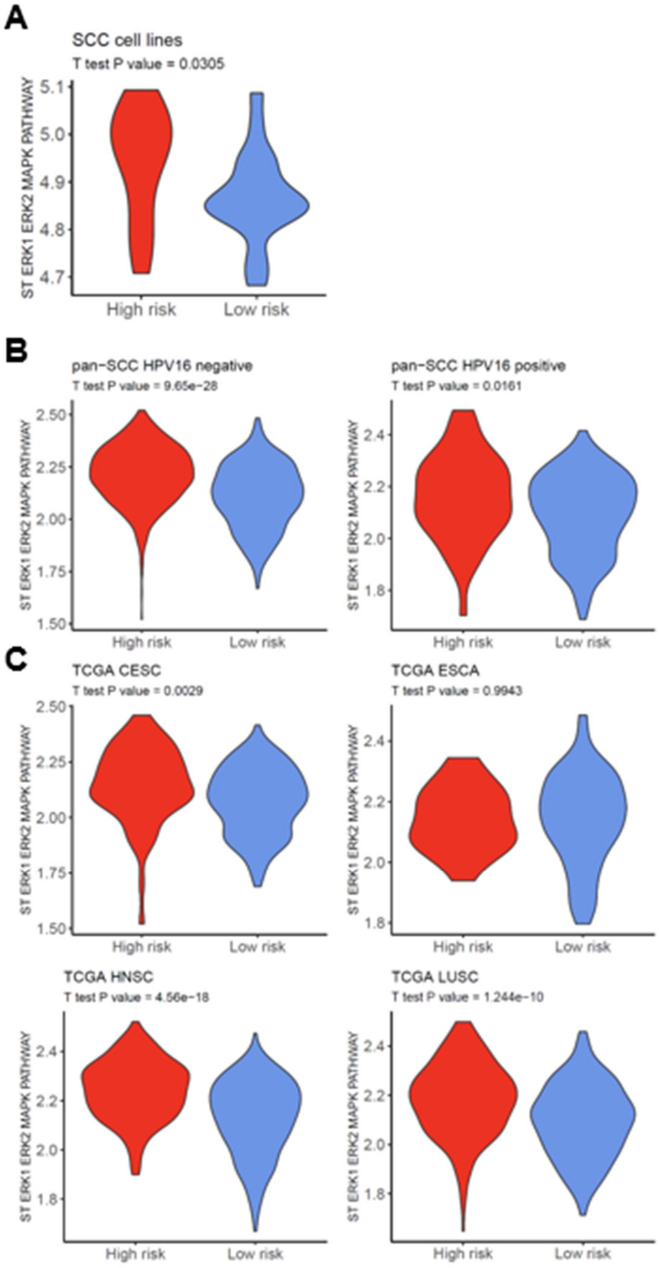

Figure S21. MAPK/ERK pathway activity in SCC cell lines and subgroups of the pan-SCC cohort. (**A**) Violin plot demonstrates a significantly higher ssGSEA score for the MAPK/ERK pathway in SCC cell lines with a high as compared to a low risk score. (**B**) Violin plots demonstrate a significantly higher ssGSEA score for the MAPK/ERK pathway in high-risk as compared to low-risk groups of HPV16-negative (left) or HPV16-positive tumors of the pan-SCC cohort. (**C**) Violin plots demonstrate a significantly higher ssGSEA score for the MAPK/ERK pathway in high-risk as compared to low-risk groups of indicated tumor types of the pan-SCC cohort except for TCGA-ESCA.

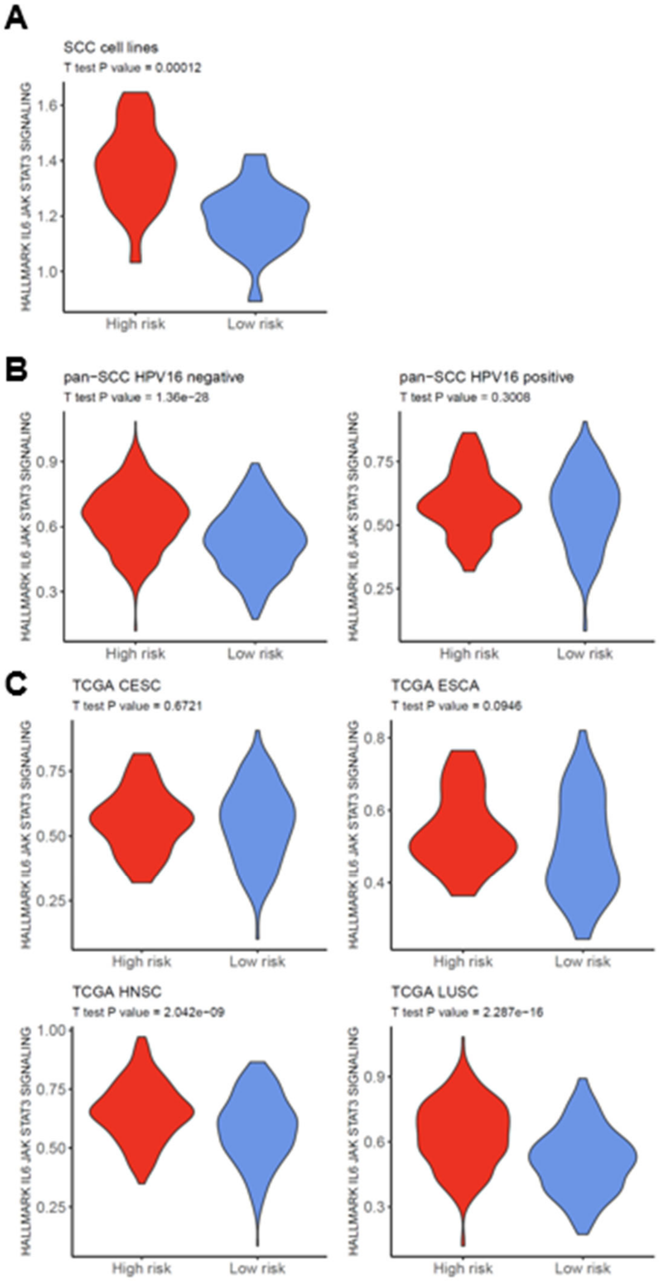

Figure S22. JAK-STAT3 pathway activity in SCC cell lines and subgroups of the pan-SCC cohort. (**A**) Violin plot demonstrates a significantly higher ssGSEA score for the JAK-STAT3 pathway in SCC cell lines with a high as compared to a low risk score. (**B**) Violin plots demonstrate a significantly higher ssGSEA score for the JAK-STAT3 pathway in high-risk as compared to low-risk groups of HPV16-negative (left) but not for HPV16-positive tumors of the pan-SCC cohort. (C) Violin plots demonstrate a significantly higher ssGSEA score for the JAK-STAT3 pathway in high-risk as compared to low-risk groups of TCGA-HNSC and TCGA-LUSC, but not for TCGA-CECA and TCGA-ESCA.

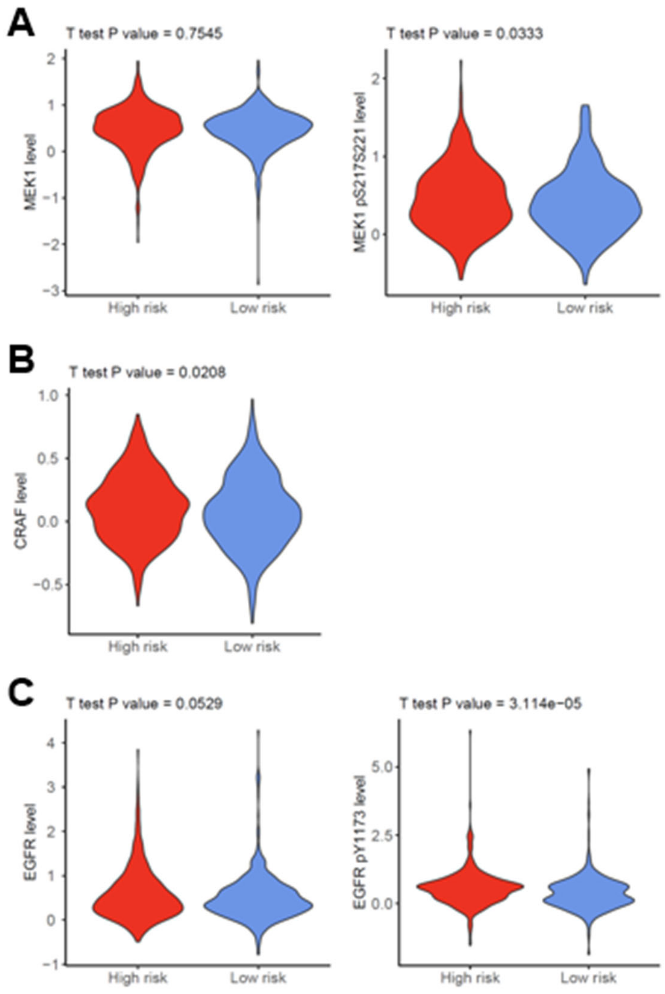

Figure S23. EGFR-RAF-MEK pathway analysis of the pan-SCC cohort according to the TCPA dataset. (**A**) Violin plots show the distribution of protein levels for MEK1 (left) and MEK1_pS217S221 (right) in tumors of the pan-SCC cohort. (**B**) Violin plot demonstrates significantly higher CRAF protein levels in the high-risk as compared to the low-risk group of the pan-SCC cohort. (**C**) Violin plots show the distribution of protein levels for EGFR (left) and EGFR_pY1173 (right) in tumors of the pan-SCC cohort.

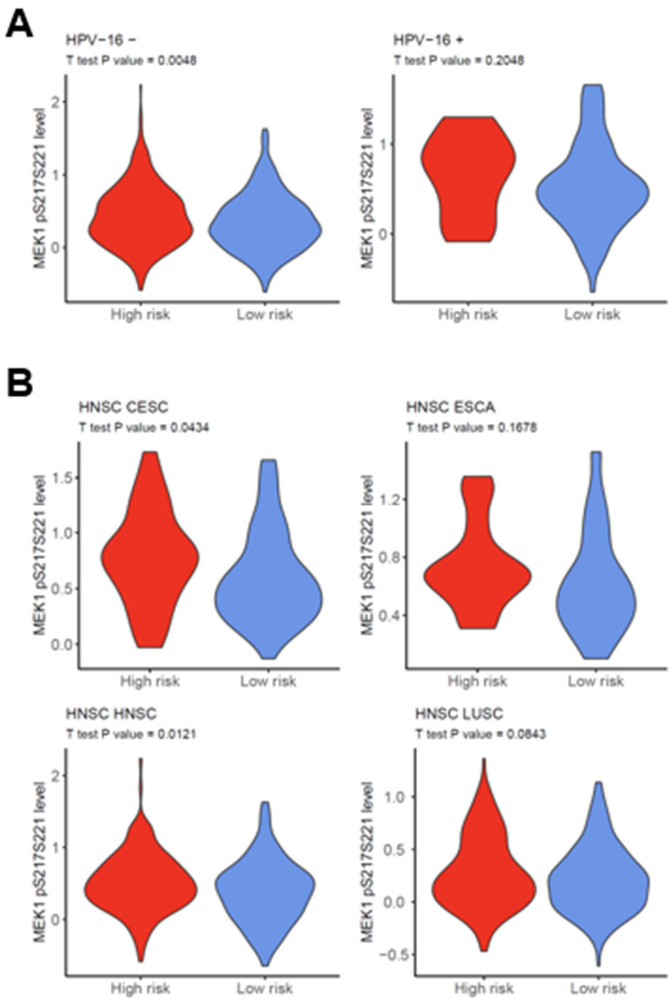

Figure S24. Subgroup analysis for MEK1 phosphorylation. (**A**) Violin plots demonstrate significantly higher MEK1_pS217S221 levels in high-risk as compared to low-risk groups for HPV16-negative (left) but not for HPV16-positive tumors of the pan-SCC cohort. (**B**) Violin plots demonstrate significantly higher MEK1_pS217S221 levels in high-risk as compared to low-risk groups for TCGA-CECA and TCGA-HNSC but not for TCGA-ESCA and TCGA-LUSC.

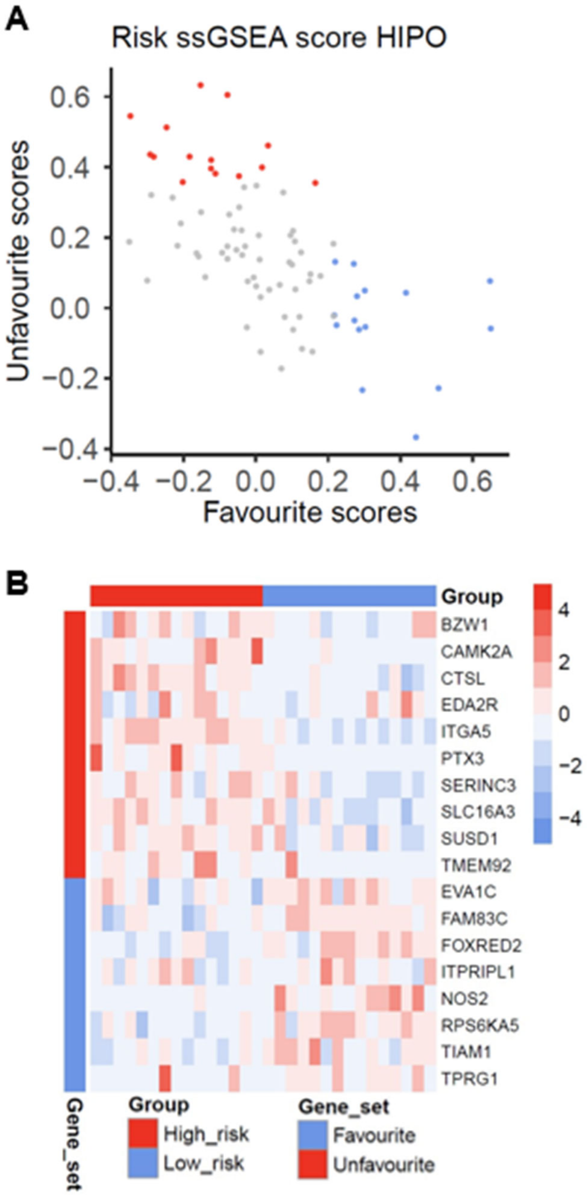

Figure S25. Risk stratification of the GSE117973 cohort. (**A**) Dot plot displays ssGSEA scores for the GSE117973 cohort based on gene sets of the 18-gene signature related to a favorable or unfavorable survival of the pan-SCC cohort. Red dots = higher association with unfavorable gene set; blue dots = higher association with favorable gene set. (**B**) Heatmap illustrates expression patterns of the18-gene signature in high-risk or low-risk groups of the GSE117973 cohort.
